# Incident cardiovascular, renal, metabolic diseases and death in individuals identified for risk-guided atrial fibrillation screening: a nationwide cohort study

**DOI:** 10.1136/openhrt-2023-002357

**Published:** 2023-07-10

**Authors:** Jianhua Wu, Ramesh Nadarajah, Yoko M Nakao, Kazuhiro Nakao, David Hogg, Keerthenan Raveendra, Ronen Arbel, Moti Haim, Doron Zahger, Campbel Cowan, Chris P Gale

**Affiliations:** 1 School of Dentistry, University of Leeds, Leeds, UK; 2 Leeds Institute of Data Analytics, University of Leeds, Leeds, UK; 3 Department of Cardiology, Leeds General Infirmary, Leeds, UK; 4 Department of Cardiovascular Medicine, National Cerebral and Cardiovascular Center, Suita, Japan; 5 School of Computing, University of Leeds, Leeds, UK; 6 Medical School, University of Leeds, Leeds, UK; 7 Sapir College, Hof Ashkelon, Israel; 8 Community Medical Services Division, Clalit Health Services, Tel Aviv, Israel; 9 Department of Cardiology, Soroka University Medical Center, Beer Sheva, Israel; 10 Ben-Gurion University of the Negev, Beer Sheva, Israel

**Keywords:** Atrial Fibrillation, PRIMARY CARE, Electronic Health Records

## Abstract

**Objective:**

Risk-guided atrial fibrillation (AF) screening may be an opportunity to prevent adverse events in addition to stroke. We compared events rates for new diagnoses of cardio-renal-metabolic diseases and death in individuals identified at higher versus lower-predicted AF risk.

**Methods:**

From the UK Clinical Practice Research Datalink-GOLD dataset, 2 January 1998–30 November 2018, we identified individuals aged ≥30 years without known AF. The risk of AF was estimated using the FIND-AF (Future Innovations in Novel Detection of Atrial Fibrillation) risk score. We calculated cumulative incidence rates and fit Fine and Gray’s models at 1, 5 and 10 years for nine diseases and death adjusting for competing risks.

**Results:**

Of 416 228 individuals in the cohort, 82 942 were identified as higher risk for AF. Higher-predicted risk, compared with lower-predicted risk, was associated with incident chronic kidney disease (cumulative incidence per 1000 persons at 10 years 245.2; HR 6.85, 95% CI 6.70 to 7.00; median time to event 5.44 years), heart failure (124.7; 12.54, 12.08 to 13.01; 4.06), diabetes mellitus (123.3; 2.05, 2.00 to 2.10; 3.45), stroke/transient ischaemic attack (118.9; 8.07, 7.80 to 8.34; 4.27), myocardial infarction (69.6; 5.02, 4.82 to 5.22; 4.32), peripheral vascular disease (44.6; 6.62, 6.28 to 6.98; 4.28), valvular heart disease (37.8; 6.49, 6.14 to 6.85; 4.54), aortic stenosis (18.7; 9.98, 9.16 to 10.87; 4.41) and death from any cause (273.9; 10.45, 10.23 to 10.68; 4.75). The higher-risk group constituted 74% of deaths from cardiovascular or cerebrovascular causes (8582 of 11 676).

**Conclusions:**

Individuals identified for risk-guided AF screening are at risk of new diseases across the cardio-renal-metabolic spectrum and death, and may benefit from interventions beyond ECG monitoring.

**WHAT IS ALREADY KNOWN ON THIS TOPIC**

- Atrial fibrillation (AF) develops in the context of other comorbidities, and individuals with AF are at risk of a range of major cardiovascular events.
- Stroke prevention has been the primary focus of AF screening research.
- The FIND-AF prediction algorithm can facilitate risk-based AF screening in the UK through primary care electronic health records. Individuals identified for risk-based screening may also be at risk of adverse events in excess of stroke.

**WHAT THIS STUDY ADDS**

- Individuals who would be identified for AF screening by FIND-AF, compared with those identified as lower risk, are at increased risk of a range of cardio-renal-metabolic adverse events, including a ≥10-fold higher hazard for heart failure, aortic stenosis and death.
- The higher-predicted risk cohort makes up less than 20% of individuals aged 30 years or older without AF, but constitutes 65% of incident aortic stenosis cases, 70% of incident heart failure cases and 71% of cardiovascular deaths over the next 10 years.

**HOW THIS STUDY MIGHT AFFECT RESEARCH, PRACTICE OR POLICY**

- Risk-based AF screening may enable targeted diagnostics or preventative strategies for eligible participants to prevent or delay adverse events beyond a narrow focus on stroke.
- Multimodal phenotyping of individuals at higher-predicted AF risk is in process to determine the burden of undiagnosed cardiovascular, renal and metabolic conditions among this cohort, and whether or not there are scalable opportunities to intervene to reduce future cardiovascular and cerebrovascular events.

Individuals who would be identified for AF screening by FIND-AF, compared with those identified as lower risk, are at increased risk of a range of cardio-renal-metabolic adverse events, including a ≥10-fold higher hazard for heart failure, aortic stenosis and death.

## Introduction

Atrial fibrillation (AF) screening research has hitherto primarily focused on stroke prophylaxis through early detection of AF and initiation of oral anticoagulation. Randomised controlled trials have demonstrated that non-invasive ECG monitoring in older people with or without stroke risk factors increases detection rates of previously undiagnosed AF compared with routine standard of care,^1–3^ but yields are relatively low (<5%) and the net benefit small.^4^

AF frequently develops due to, and in parallel with, other cardiovascular, renal and metabolic conditions.^5^ Over 70% of new diagnoses have at least two concomitant, chronic comorbidities,^6^ and thereafter are at an increased risk of major cardiovascular events beyond stroke, including ischaemic heart disease, heart failure, chronic kidney disease, peripheral vascular disease and death.^7^

Risk-guided AF screening has the potential to achieve a higher yield of AF detection than age-guided screening.^8^ Furthermore, individuals identified at elevated risk of AF may have an age and comorbidity profile similar to individuals with diagnosed AF, and thus also be at risk of subsequent adverse events. If so, a risk-guided AF screening strategy may provide an opportunity for the identification and management of concomitant diseases and cardiometabolic risk factors to prevent a range of adverse events beyond stroke.^5^

To determine whether individuals identified for risk-guided AF screening are at increased risk of adverse events, we used a large nationwide longitudinal database of linked primary and secondary care records to study event rates in the subpopulation at higher-predicted AF risk for a range of new-onset cardio-renal-metabolic diseases and death.

## Methods

### Data source

We used electronic health records (EHRs) from the Clinical Practice Research Datalink (CPRD) from 1 January 1985 to 30 November 2018. The CPRD database contains anonymised patient data from approximately 7% of the UK population and is broadly representative in terms of age, sex and ethnicity.^9^ CPRD is one of the world’s largest databases of longitudinal medical records from primary care. The dataset used for this analysis was primary care records from CPRD that had been linked to secondary care admission records from Hospital Episodes Statistics Admitted Patient Care data and death certificates from the Office for National Statistics (ONS). Linkage is available for a subset of English practices from 1 January 1998, covering approximately 50% of all CPRD records. Previous research has demonstrated the representativeness of patients eligible for linkage in terms of age, sex and geography.^10^ More than 200 independent studies have investigated the validity of diagnoses recorded in CPRD, which reported an average positive predictive value of about 90% for a broad range of conditions.^11^

### Study population

We included adults registered at practices within CPRD who were ≥30 years of age at entry with no history of AF and at least 1-year follow-up, between 2 January 1998 and 30 November 2018. All individuals were categorised as lower or higher-predicted AF risk by the FIND-AF risk score,^8^ with the higher-risk cohort reflecting individuals who would be identified for risk-guided AF screening.

The FIND-AF risk score predicts incident AF at 6 months for individuals ≥30 years of age without a preceding diagnosis of AF.^8^ The risk score is scalable through community-based EHRs because it only requires data for age, sex, comorbidities and ethnicity (included an ‘ethnicity unrecorded’ category where it was unavailable because missingness was considered to be informative; online supplemental table 1).^12^ The risk score was found to have stronger discriminative performance, reclassification and net benefit for short-term incident AF than the CHA_2_DS_2_-VASc and C_2_HEST scores, and more efficiently identifies individuals who develop AF than an age-guided approach.^8^

10.1136/openhrt-2023-002357.supp1 Supplementary data

### Outcomes

The primary endpoint for the analysis was the initial presentation of a cardiovascular, renal, or metabolic disease or death. To best characterise highly prevalent and morbid diseases, associated with the development or consequence of AF (online supplemental figure 1),^5^ we individually examined the following nine conditions: heart failure, valvular heart disease (and specifically aortic stenosis), myocardial infarction, stroke (ischaemic and haemorrhagic) or transient ischaemic attack, peripheral vascular disease, chronic kidney disease, diabetes mellitus, as well as chronic obstructive pulmonary disease (COPD). We also investigated for occurrence of death by any cause recorded in primary care or by death certification from the UK Death Register of the ONS, which was mapped on to nine disease categories (online supplemental table 2). For each condition, a list of diagnostic codes from the CALIBER code repository, including from International Classification of Diseases 10th revision (used in secondary care) and Read coding schemes (used in primary care), was defined to comprehensively identify diagnoses from EHRs (online supplemental table 3). Incident diagnoses were defined as the first record of that condition in primary or secondary care records from any diagnostic position. For definition of new cases, we excluded individuals for the analysis of each condition who had a diagnosis of that condition before the patient’s entry to the study. If no indication of a specific disease was recorded, then the patient was assumed to be free from the disease.

### Statistical analysis

The baseline characteristics are summarised by predicted AF status. Continuous variables were reported as mean±SD. Categorical variables were reported as frequencies with corresponding percentages.

We created Kaplan-Meier plots for individuals identified as higher and lower-predicted risk of AF and derived the cumulative incidence rate for each outcome at 1, 5 and 10 years considering the competing risk of death, as well as death at 5 and 10 years. For each specified outcome, we calculated the HR between higher and lower-predicted risk of AF using the Fine and Gray’s model with adjustment for the competing risk of death. We reported unadjusted HR and adjusted HR where the model was adjusted for age, sex, ethnicity and the presence of any of the other outcomes at baseline.

Given that age and sex were two key variables in the FIND-AF algorithm,^8^ and some of the outcomes have incidence rates that are strongly associated with age (eg, aortic stenosis) or differ by sex (eg, heart failure),^13 14^ we conducted subgroup analyses of incidence rates for higher and lower-risk individuals for each outcome by age group (30–64 years and ≥65 years) and sex. As some of the outcomes are more likely to occur in the setting of prevalent AF (eg, stroke or heart failure),^5^ we also conducted a sensitivity analysis where people with incident AF during follow-up were excluded.

Study findings are reported in accordance with the Reporting of studies Conducted using Observational Routinely-collected health Data recommendations,^15^ and the CODE-EHR best-practice framework for using structured electronic healthcare records in clinical research.^16^ We used R V.4.1.0 for all analyses.

### Patient and public involvement

The Arrhythmia Alliance, an AF association, provided input on the FIND-AF scientific advisory board. The FIND-AF patient and public involvement group have given input to reporting and dissemination plans of the research.

## Results

### Patient population

In the cohort of 416 228 individuals (average age 49.9 (SD 15.4) years, 50.8% women, 86.8% white), 82 942 (19.9%) were identified as higher-predicted risk of AF, 3483 of whom were <65 years of age, with 1203 and 8876 diagnosed with AF over 6 months and 10 years of follow-up, respectively. At point of risk prediction, those at higher compared with lower-predicted AF risk had a higher average age and prevalence of baseline comorbidities (table 1). The cohort with higher-predicted AF risk had similar baseline characteristics and mean CHA_2_D_2_2-VASc score to the cohort who developed AF during follow-up, but a lower prevalence of ischaemic heart disease (15.1% vs 20.2%), prior stroke or transient ischaemic attack (7.7% vs 12.2%), hypertension (35.7% vs 40.0%), valvular heart disease (1.7% vs 5.4%) and chronic kidney disease (3.6% vs 6.4%) (online supplemental table 4).

**Table 1:** Baseline characteristics of analytical cohort stratified by predicted AF risk

	FIND-AF predicted risk	
	Lower risk n (%)	Higher risk n (%)
	333 286	82 942
**Demographics**		
Age, years	44.1 (10.40)	73.2 (8.75)
Sex (women)	170 568 (51.2)	41 210 (49.7)
Ethnicity		
Asian	7385 (2.2)	894 (1.1)
Black	5786 (1.7)	613 (0.7)
Other	22 033 (6.6)	5878 (7.1)
Unknown	91 505 (27.5)	2161 (2.6)
White	206 577 (62.0)	73 396 (88.5)
**Comorbidities**		
Anaemia	9118 (2.7)	4251 (5.1)
Aortic stenosis	63 (<0.1)	316 (0.4)
Cancer	6120 (1.8)	8303 (10.0)
COPD	1111 (0.3)	4019 (4.8)
Chronic kidney disease	2938 (0.9)	2990 (3.6)
Diabetes mellitus	6328 (1.9)	8072 (9.7)
Dyslipidaemia	6095 (1.8)	5984 (7.2)
Ischaemic heart disease	3 299 (1.0)	12 486 (15.1)
Heart failure	163 (<0.1)	2 748 (3.3)
Hypertension	20 139 (6.0)	29 594 (35.7)
Hyperthyroidism	1883 (0.6)	1370 (1.7)
Stroke/TIA	1376 (0.4)	6375 (7.7)
Valvular heart disease	562 (0.2)	1414 (1.7)

AF, atrial fibrillation; COPD, chronic obstructive pulmonary disease; TIA, transient ischaemic attack.

### Outcomes

**Figure 1:**
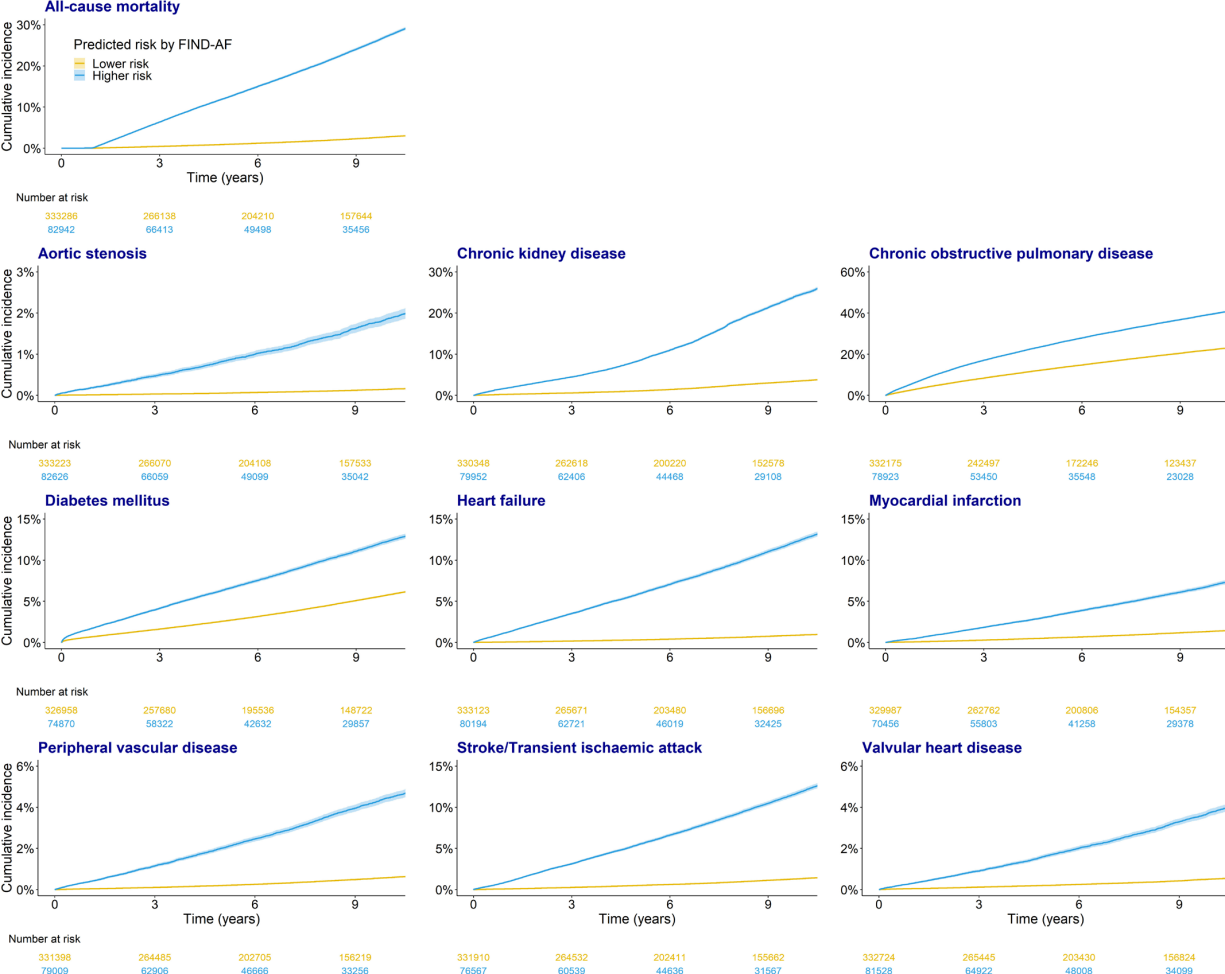
Kaplan-Meier plots for the 10 outcomes. AF, atrial fibrillation.

Higher-predicted AF risk, compared with lower-predicted AF risk, was associated with increased occurrence for each prespecified condition at 1, 5 and 10 years of follow-up (figure 1 and table 2).

**Table 2:** Cumulative incidence rate for the 10 outcomes stratified by predicted AF risk

Outcome	Median time to event (years, IQR)		Cumulative incidence (per 1000 persons)					
	Predicted lower risk	Predicted higher risk	Predicted lower risk			Predicted higher risk		
			1 year	5 years	10 years	1 year	5 years	10 years
Aortic stenosis	5.23 (2.45–7.81)	4.41 (1.98–7.18)	0.1 (0.1–0.2)	0.5 (0.4–0.6)	1.5 (1.3–1.7)	1.6 (1.4–1.9)	8.2 (7.5–8.9)	18.7 (17.5–19.9)
COPD	3.12 (1.28–5.84)	2.68 (1.11–5.31)	32.2 (31.6–32.8)	127.4 (126.2–128.6)	222.2 (220.4–223.9)	68.4 (66.6–70.2)	244.6 (241.4–247.8)	395.8 (391.5–400.0)
Chronic kidney disease	5.95 (3.03–7.83)	5.44 (2.76–7.60)	2.3 (2.1–2.4)	10.6 (10.2–11.0)	35.3 (34.5–36.1)	17.4 (16.5–18.4)	82.9 (80.8–85.0)	245.2 (241.3–249.1)
Diabetes mellitus	4.24 (1.62–7.10)	3.45 (1.30–6.33)	7.2 (6.9–7.4)	26.1 (25.5–26.7)	57.9 (56.9–58.9)	17.9 (16.9–18.8)	64.4 (62.5–66.3)	123.3 (120.4–126.3)
Heart failure	5.49 (2.71–7.89)	4.06 (1.82–6.84)	0.6 (0.5–0.6)	3.1 (2.9–3.3)	9.0 (8.6–9.4)	11.9 (11.2–12.7)	58.3 (56.5–60.1)	124.7 (121.7–127.6)
Myocardial infarction	4.95 (2.54–7.50)	4.32 (2.03–6.88)	0.9 (0.8–1.0)	5.4 (5.1–5.7)	13.6 (13.1–14.1)	5.5 (5.0–6.1)	31.4 (30.0–32.8)	69.6 (67.2–72.0)
Peripheral vascular disease	5.59 (2.83–7.83)	4.28 (2.05–6.96)	0.4 (0.3–0.4)	2.0 (1.8–2.1)	5.8 (5.5–6.2)	3.7 (3.3–4.2)	20.1 (19.1–21.2)	44.6 (42.8–46.4)
Stroke/TIA	5.17 (2.63–7.79)	4.27 (2.01–6.92)	0.8 (0.7–0.9)	5.0 (4.7–5.2)	13.3 (12.8–13.8)	9.2 (8.6–9.9)	54.1 (52.4–55.9)	118.9 (116.0–121.8)
Valvular heart disease	4.89 (2.25–7.72)	4.54 (2.12–7.11)	0.5 (0.4–0.5)	2.0 (1.9–2.2)	5.2 (4.8–5.5)	3.0 (2.6–3.4)	16.3 (15.4–17.3)	37.8 (36.1–39.5)
All-cause mortality	5.72 (3.24–8.06)	4.75 (2.66–7.27)		9.2 (8.8–9.6)	27.9 (27.2–28.6)		121.6 (119.2–124.0)	273.9 (270.2–277.5)

AF, atrial fibrillation; COPD, chronic obstructive pulmonary disease; TIA, transient ischaemic attack.

A quarter of individuals in the higher-predicted AF risk cohort were diagnosed with COPD within 5 years and with chronic kidney disease within 10 years. Furthermore, within 10 years each of heart failure, diabetes mellitus and stroke or transient ischaemic attack were diagnosed in more than 10% of individuals at higher-predicted AF risk. Relative to individuals at lower-predicted AF risk, those with higher-predicted AF risk were at 12.54-fold (95% CI 12.08 to 13.01) increased risk of heart failure, 9.98-fold increased risk of aortic stenosis (95% CI 9.16 to 10.87) and 8.07-fold increased risk of stroke/transient ischaemic attack (95% CI 7.80 to 8.34) (table 3).

**Table 3:** HRs for incident outcomes among individuals at higher-predicted AF risk compared with individuals at lower-predicted AF risk

Outcome	Events/cohorts		Unadjusted HR (95% CI)	Adjusted HR (95% CI)
	Lower risk	Higher risk		
Aortic stenosis	851/333 223	1557/82 626	9.98 (9.16 to 10.87)	1.64 (1.43 to 1.87)
COPD	66 941/332 175	27 110/78 923	2.02 (2.00 to 2.05)	1.17 (1.14 to 1.20)
Chronic kidney disease	15 077/33 0348	17 494/79 952	6.85 (6.70 to 7.00)	1.46 (1.41 to 1.51)
Diabetes mellitus	21 627/326 958	8338/74 870	2.05 (2.00 to 2.10)	1.06 (1.02 to 1.10)
Heart failure	4135/333 123	9453/80 194	12.54 (12.08 to 13.01)	1.63 (1.54 to 1.73)
Myocardial infarction	5111/329 987	4483/70 456	5.02 (4.82 to 5.22)	1.09 (1.03 to 1.17)
Peripheral vascular disease	2470/331 398	3176/79 009	6.62 (6.28 to 6.98)	1.30 (1.19 to 1.42)
Stroke/TIA	5884/331 910	8573/76 567	8.07 (7.80 to 8.34)	1.40 (1.33 to 1.48)
Valvular heart disease	2426/332 724	2946/81 528	6.49 (6.14 to 6.85)	1.56 (1.43 to 1.71)
All-cause mortality	12 804/333 286	25 814/82 942	10.45 (10.23 to 10.68)	1.06 (1.02 to 1.09)

Model was adjusted for age, sex, ethnicity and the presence of any of the other outcomes at baseline.

AF, atrial fibrillation; COPD, chronic obstructive pulmonary disease; TIA, transient ischaemic attack.

The higher-predicted AF risk cohort was also more than five times more likely to be diagnosed with chronic kidney disease, valvular heart disease, myocardial infarction and peripheral vascular disease, and twice as likely to experience COPD or diabetes mellitus. Furthermore, the median time to event was shorter for each outcome in the higher-predicted risk cohort compared with the lower-predicted risk cohort, with a difference of over a year for heart failure (4.06 vs 5.49) and peripheral vascular disease (4.28 vs 5.59).

Death was common among persons identified as higher-predicted AF risk, with over a quarter of patients having died by 10 years (table 2). On unadjusted analysis, individuals at higher-predicted AF risk were at 10.5-fold increased hazard for death compared with individuals at lower-predicted AF risk (95% CI 10.23 to 10.68; table 3). Of the 25 814 deaths during 10-year follow-up in the higher-predicted AF risk cohort, 8582 (33%) were as a result of cardiovascular disease or cerebrovascular disease, with 5931 (23%) attributed to cancer (table 4).

**Table 4:** Cause of death stratified by FIND-AF risk classification

	Predicted AF risk	
	Lower risk	Higher risk
	n=333 286	n=82 942
Cause of death		
Cardiovascular disease	2506 (0.8)	6006 (7.2)
Cerebrovascular disease	588 (0.2)	2576 (3.1)
Chronic respiratory disease	751 (0.2)	1952 (2.4)
Digestive disease	701 (0.2)	1125 (1.4)
Infection	573 (0.2)	2531 (3.1)
Injuries	494 (0.1)	471 (0.6)
Kidney disease	43 (0.0)	233 (0.3)
Mental and neurological disease	546 (0.2)	2144 (2.6)
Neoplasms	4889 (1.5)	5931 (7.2)

AF, atrial fibrillation.

During the 10-year follow-up, 70% of incident heart failure cases (9453 of 13 588), and 65% of incident aortic stenosis diagnoses (1557 of 2408) occurred in individuals at higher-predicted AF risk, even though they only accounted for less than one-fifth of the total cohort. Of the 38 618 deaths that occurred during follow-up, two-thirds occurred in the higher-predicted AF risk cohort (25 814; 67%). Specifically, individuals in the higher-predicted AF risk cohort constituted three-quarters of the deaths related to cardiovascular or cerebrovascular disease (8582 of 11 676; 74%), whereas the burden of death from neoplasm was more evenly distributed between individuals at lower and higher-predicted AF risk (total deaths attributed to neoplasm 10 820; deaths in lower-predicted AF risk cohort 4889 (45%); deaths in higher-predicted AF risk cohort 5931 (55%)).

### Subgroup analysis

On subgroup analysis, higher-predicted AF risk, compared with lower-predicted AF risk, was associated with increased incidence for each of the outcomes in both men and women and in younger (age 30–64 years) and older (age ≥65 years) individuals (online supplemental figures 2–5). Excluding patients with incident AF during follow-up did not change the direction or magnitude of events (online supplemental table 5).

**Figure 2:**
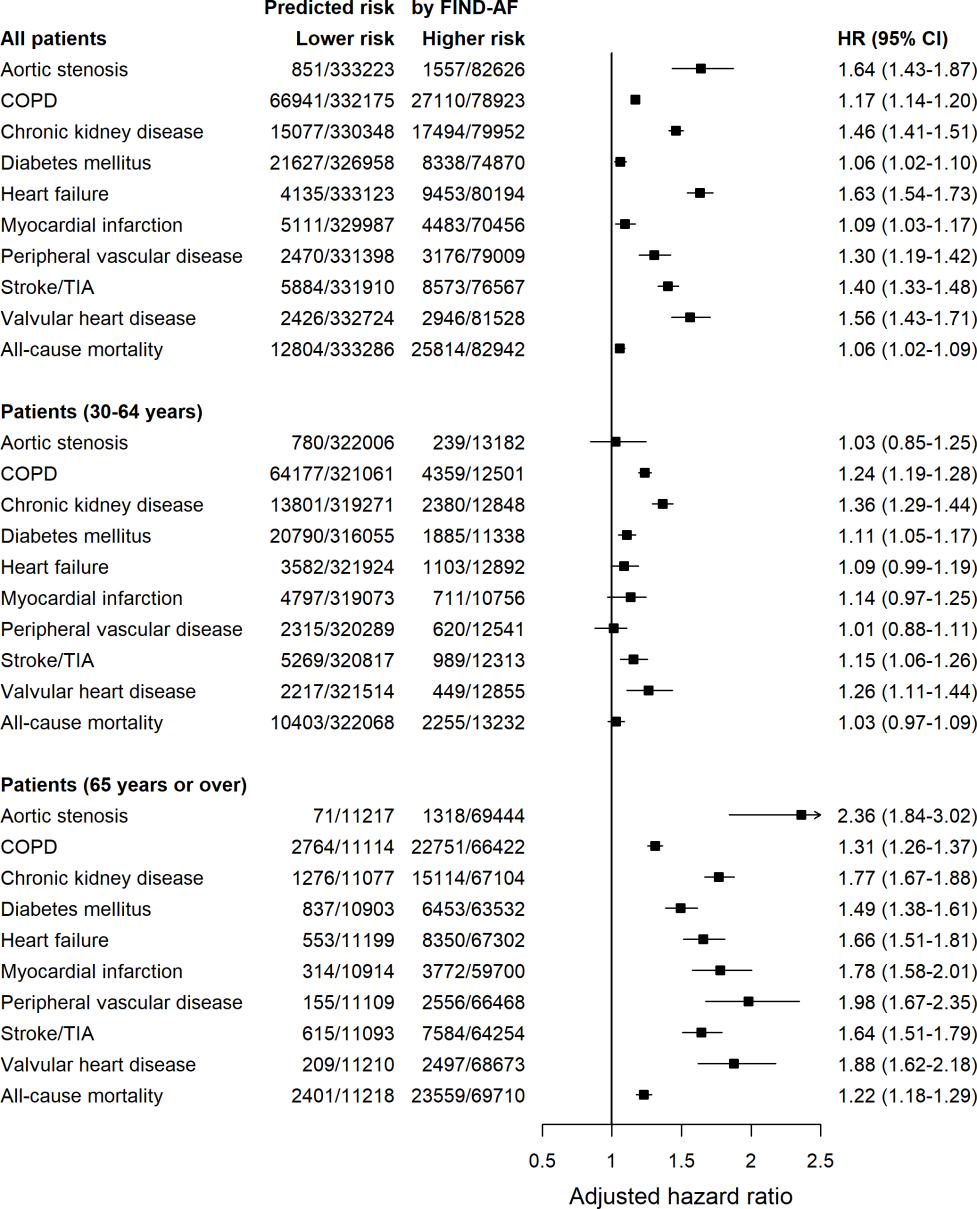
Adjusted HRs for the 10 outcomes, stratified by age. HRs among individuals at higher-predicted AF risk compared with individuals at lower-predicted AF risk for the 10 outcomes when adjusted for age, sex, ethnicity and the presence of any of the other outcomes at baseline. AF, atrial fibrillation; COPD, chronic obstructive pulmonary disease; TIA, transient ischaemic attack.

After adjustment for age, sex, ethnicity and presence of any other outcomes at baseline, higher-predicted AF risk remained associated with excess risk for all-cause death and each condition (figure 2 and table 3). The magnitude of independent associations was greater in older compared with younger individuals. It was highest for aortic stenosis, followed in descending order by peripheral vascular disease, valvular heart disease, myocardial infarction, chronic kidney disease, heart failure, stroke or transient ischaemic attack, diabetes mellitus, COPD and death.

## Discussion

**Figure 3:**
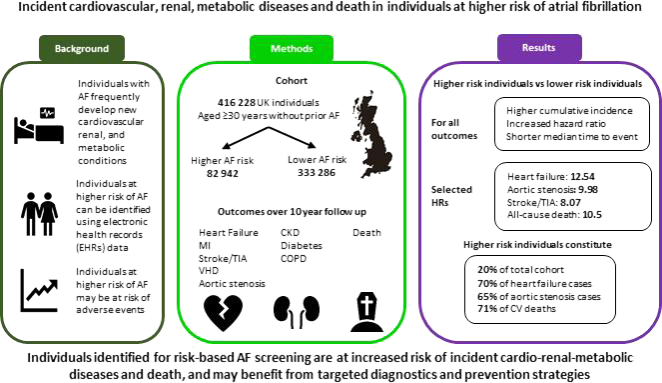
Incident cardiovascular, renal and metabolic diseases and death for individuals at higher risk of atrial fibrillation (AF). CKD, chronic kidney disease; COPD, chronic obstructive pulmonary disease; CV, cardiovascular; MI, myocardial infarction; TIA, transient ischaemic attack; VHD, valvular heart disease.

In this population-based study, we found that individuals identified for risk-guided AF screening had a similar age and comorbidity profile to individuals who develop AF, and were at increased risk of a range of cardiovascular, renal, and metabolic diseases and death (figure 3). Over a decade of follow-up, more than a quarter of individuals at higher-predicted AF risk received a new diagnosis of chronic kidney disease, with heart failure and diabetes mellitus diagnosed in more than 1 in 10. Although the higher-predicted AF risk cohort only made up one-fifth of the total population, it constituted 70% of new heart failure diagnoses and 65% of new aortic stenosis diagnoses. The risk of death from any cause was 10-fold greater for individuals at higher-predicted AF risk, who accounted for two-thirds of deaths observed during follow-up, and three-quarters of the deaths attributed to cardiovascular or cerebrovascular disease. Adjusted analysis demonstrated that AF risk was associated with incident diseases and death beyond advanced age, which has been the predominant approach hitherto used in AF screening research and advocated in guidelines.^4 5^

Elevated AF risk portended incident diseases across the cardio-renal-metabolic axis, including when incident AF cases during follow-up were excluded. Structural and electric remodelling of the atrium, which increases AF susceptibility, is contributed to by a continuum of unhealthy lifestyle, risk factors and comorbidities^17^; and systemic inflammation, myocardial ischaemia and autonomic dysfunction are implicated in AF genesis.^17^ Age, smoking, obesity, inflammatory diseases and hypertension are shared risk factors between AF, vascular disease, aortic stenosis, heart failure, diabetes mellitus and chronic kidney disease.^18–20^ Aortic stenosis and heart failure share neurohormonal and proinflammatory pathways with AF which induce myocardial inflammation and fibrosis.^17 21^ Thus, AF is not a disease process in isolation, but a manifestation of multisystem pathology—and AF risk may be considered a precursor stage for an AF ‘syndrome’ of clustered disease states.

Previous studies of AF risk have only investigated for occurrences of AF and stroke during follow-up, reflecting a narrower focus on stroke prevention through early AF detection and treatment.^22^ Increasingly, it is recognised that the majority of individuals with AF are older and/or have a higher burden of concomitant diseases, cardiometabolic risk factors and unhealthy lifestyle behaviours.^5^ Accordingly, lifestyle interventions and management of specific cardiovascular risk factors/comorbidities are recommended in contemporary guidelines for patients with newly diagnosed AF.^5^ People identified for risk-guided AF screening share the same characteristics as those with AF, so they may also benefit from equivalent interventions.

Our findings suggest that a risk-guided approach to AF screening may present an opportunity to intervene beyond AF detection and prescription of oral anticoagulation for stroke prophylaxis. The UK National Health Service Health Check aims to prevent stroke and cardiovascular disease at a cost £165 million per year,^23^ but includes a population comprising only 20% of all strokes and myocardial infarction.^24^ By contrast, the higher-predicted AF risk subpopulation experience the majority of incident heart failure and vascular events, as well as cardiovascular and cerebrovascular deaths. Based on our findings, risk-guided AF screening would be offered to a subpopulation of 339 000 people aged ≤65 years in the UK, and of this cohort, 20% and 15% developed new chronic kidney disease and diabetes mellitus, respectively, over the next 10 years (online supplemental figure 2). The median time to event for these outcomes was in excess of 3 years, so it may be appropriate to offer this ‘targeted’ group comprehensive programmes designed to improve risk factor profiles,^9^ as well as early initiation of therapeutics such as sodium-glucose cotransporter 2 inhibitors to reduce the risk of disease progression and cardiovascular morbidity.^25–27^ Furthermore, older persons identified for AF screening were more than twice as likely to be diagnosed with aortic stenosis as their lower-risk counterparts. Thus, this cohort may benefit from targeted early diagnostics, which may not be effective and cost-effective in a purely age-guided AF screening cohort. Elevated natriuretic peptide levels may similarly uncover the presence of underlying multisystemic or structural cardiac changes, and has been demonstrated to increase the yield of AF screening,^28^ but employing wide-scale natriuretic peptide testing would be resource-intensive. Biomarker testing may be more efficiently employed as part of a stepwise approach after risk assessment.

Treatment for individuals at risk of heart failure has been demonstrated to improve outcomes,^29^ and accordingly collaborative care for individuals at risk of AF may reduce the subsequent incidence of AF and other adverse events. To prospectively determine the burden of undiagnosed or undertreated cardiovascular, renal and metabolic conditions and risk factors in individuals identified for risk-guided AF screening, participants enrolled in the FIND-AF pilot implementation study (The British Heart Foundation Bristol Myers Squibb Cardiovascular Catalyst Award–CC/22/250026) will undergo biomarker and imaging characterisation and cardiologist review, with long-term digital follow-up for the outcomes investigated here.

There are some limitations to our study. First, the CPRD database is routinely collected; retrospective primary care data and underestimation of incidence of outcomes in this study are possible, since there will have been individuals with unrecorded diagnoses. Second, incomplete clinical information is contained in available structured data from EHRs. In particular, echocardiographic reports were unavailable for left ventricular ejection fraction or valve disease severity. Consequently, we could not differentiate types of heart failure, though all are associated with increased risk of death and hospitalisation.^13^ We were also unable to provide evidence for the proportion of aortic stenosis cases that were eligible for intervention. However, aortic stenosis is a progressive condition, so we considered an increased risk of clinical diagnosis as important.^14^ Third, it is possible that AF risk is associated with increased risk of diseases outside of those we investigated (for example, different cancers). Here we sought to assess association with diseases where there was an underlying pathophysiological rationale and available treatment options,^5^ rather than take a data-driven approach. Fourth, our cohort was risk stratified at a single time point, in keeping with how AF screening would be implemented in practice, and we did not address changes in risk profile over time. Fifth, this study included a UK-based cohort and the association between predicted AF risk and incident diseases and death in other geographies may vary. Sixth, individuals for risk-guided AF screening were identified by the FIND-AF risk score, which is scalable in European community-based EHRs and has demonstrated better prediction performance for incident AF than other scalable risk scores.^8^ It seems likely that elevated AF risk calculated from other AF risk scores would be associated with incident cardio-renal-metabolic diseases and death, but the magnitude of association may vary.

## Conclusions

Individuals identified for risk-guided AF screening are also at higher risk of new diseases across the cardio-renal-metabolic spectrum and death. Participants in risk-guided AF screening may benefit from targeted diagnostics and prevention strategies in excess of ECG monitoring for AF detection.

## Data Availability

Data may be obtained from a third party and are not publicly available. Data used in this study can be accessed through CPRD subject to protocol approval. The algorithm can be shared with researchers who agree to use it only for research purposes with a data sharing agreement.
