# Supplementary material for: Incident cardiovascular, renal, metabolic diseases and death in individuals identified for risk-guided atrial fibrillation screening: a nationwide cohort study
Source: Open Heart. 2023 Jul 10;10(2):e002357. doi: 10.1136/openhrt-2023-002357 (PMC10335593; doi:10.1136/openhrt-2023-002357)
Supplement: Supplementary data [file openhrt-2023-002357supp001.pdf]

Supplemental Material

Incident cardiovascular, renal, metabolic diseases and death in individuals identified for risk-guided atrial fibrillation screening: a nationwide cohort study

Jianhua Wu*, Ramesh Nadarajah*, Yoko M Nakao, Kazuhiro Nakao, David C Hogg, Keerthenan Raveendra, Ronen Arbel, Moti Haim, Doron Zahger, Campbell Cowan, Chris P Gale

Supplementary Methods 2
Supplementary Table 1. Variables included in the FIND-AF algorithm 2
Supplementary Table 2. Definition of disease categories for causes of deaths 6
Supplementary Table 3. Read codes and ICD-10 codes used to define the outcomes 7
Supplementary Figure 1. Study design process leading to selection of study outcomes 40
Supplementary Results 41
Supplementary Table 4. Baseline characteristics of testing set, stratified by incident AF and predicted AF risk 41
Supplement Table 5. Cumulative incidence rate for the 10 outcomes at 1, 5, and 10 years of follow up stratified by predicted AF risk, when incident AF cases are excluded 43
Supplementary Figure 2. Kaplan-Meier plots for incident outcomes in individuals aged 30-64 years at baseline 44
Supplementary Figure 3. Kaplan-Meier plots for incident outcomes in individuals aged ≥65 years at baseline 46
Supplementary Figure 4. Kaplan-Meier plots for incident outcomes for men 48
Supplementary Figure 5. Kaplan-Meier plots for incident outcomes for women 49

Wu J, *et al. Open Heart* 2023; 10:e002357. doi: 10.1136/openhrt-2023-002357

Supplementary Methods

Supplementary Table 1. Variables included in the FIND-AF algorithm

**Comorbidity associated with / predictive of atrial fibrillation**	**Categorisation**	**References and Rationale for categorisation**
Demographics		
Age	-	Hindricks G, Potpara T, Dagres N, et al. 2020 ESC Guidelines for the diagnosis and management of atrial fibrillation developed in collaboration with the European Association of Cardio-Thoracic Surgery (EACTS). Eur Heart J 2020<br><br>Incidence of AF increases with age (therefore included as a continuous variable)
Sex	Men	Hindricks G, Potpara T, Dagres N, et al. 2020 ESC Guidelines for the diagnosis and management of atrial fibrillation developed in collaboration with the European Association of Cardio-Thoracic Surgery (EACTS). Eur Heart J 2020<br><br>AF is more common in men
	Women	
Ethnicity	Asian	Shen AY-J, Contreras R, Sobnosky S, et al. Racial/ethnic differences in the prevalence of atrial fibrillation among older adults—a cross-sectional study. J Natl Med Assoc 2010;102(10):906-14.<br><br>Chiang C-E, Zhang S, Tse HF, et al. Atrial fibrillation management in Asia: from the Asian expert forum on atrial fibrillation. Int J Cardiol 2013;164(1):21-32.<br><br>White, Asian, pacific Asian, and black ethnicities have different odds ratios of development of AF
	Black	
	Mixed	
	Other	
	Pacific Asian	
	White	
Alcohol use	Ex-	Samokhvalov AV, Irving HM, Rehm J. Alcohol consumption as a risk factor for atrial fibrillation: a systematic review and meta-analysis. European Journal of Preventive Cardiology 2010;17(6):706-12.<br><br>There is a monotonic dose-response relationship between alcohol consumption and AF incidence
	Light,	
	Moderate	
	Excess	
	Unspecified	
Smoking	Current	Heeringa J, Kors JA, Hofman A, et al. Cigarette smoking and risk of atrial fibrillation: the Rotterdam Study. Am Heart J 2008;156(6):1163-69.<br><br>Watanabe I. Smoking and risk of atrial fibrillation: Elsevier, 2018.<br><br>Current and ex-smokers are at increased risk of AF, with a higher risk in current smokers.
	Ex	
Weight	Obese	See table S4
	Overweight	
	Under-weight	
Comorbidities		
Adult congenital heart disease	-	-
Anaemia	-	-

Cancer	Leukaemia	Thompson PA, Lévy V, Tam CS, et al. Atrial fibrillation in CLL patients treated with ibrutinib. An international retrospective study. Br J Haematol 2016;175(3):462-66.<br><br>Sorigue M, Gual-Capllonch F, Garcia O, et al. Incidence, predictive factors, management, and survival impact of atrial fibrillation in non-Hodgkin lymphoma. Ann Hematol 2018;97(9):1633-40.<br><br>Han H, Chen L, Lin Z, et al. Prevalence, trends, and outcomes of atrial fibrillation in hospitalized patients with metastatic cancer: findings from a national sample. Cancer medicine 2021;10(16):5661-70.<br><br>AF risk is higher in patients with leukaemia and lymphoma, especially treated with iritunib. Solid organ cancers (such as lung and colorectal cancer) are more likely to undergo surgery. Metastatic disease is associated with higher risk of AF compared to non-metastatic disease. Skin cancers other than melanoma have a lower risk of metastasis and hence AF.
	Lymphoma	
	Metastasis	
	Skin cancers other than melanoma	
	Solid organ	
Cardiac surgery	Valvular,	See table S4
	Non-valvular	
Chronic kidney disease	Stage 1-2	Alonso A, Lopez FL, Matsushita K, et al. Chronic kidney disease is associated with the incidence of atrial fibrillation: the Atherosclerosis Risk in Communities (ARIC) study. Circulation 2011;123(25):2946-53.<br><br>Risk of AF increases as CKD stage worsens and if there is proteinuria
	Stage 3	
	Stage 4	
	Stage 5	
	Unspecified	
	Other	
COPD	-	-
Cerebro-vascular accident	Intracerebral haemorrhage	Hindricks G, Potpara T, Dagres N, et al. 2020 ESC Guidelines for the diagnosis and management of atrial fibrillation developed in collaboration with the European Association of Cardio-Thoracic Surgery (EACTS). Eur Heart J 2020<br><br>Association with AF is higher for ischaemic strokes than haemorrhagic strokes
	Subarachnoid haemorrhage	
	Unspecified	
Diabetes Mellitus	Good control	Dublin S, Glazer NL, Smith NL, et al. Diabetes mellitus, glycemic control, and risk of atrial fibrillation. J Gen Intern Med 2010;25(8):853-58.<br><br>Poorer glycaemic control is associated with a higher risk of AF compared to better glycaemic control or no diabetes
	Poor control	
	Unspecified / secondary	
Deep venous thrombosis	-	-
Dyslipidaemia	-	-
Infective Endocarditis	-	-
Electrophysiology procedure affecting the atria	-	-
Gout	-	-
Hypertrophic cardiomyopathy	-	-
Heart failure	-	-
Hypertension	Poor control	

	Unspecified / secondary	Dzeshka MS, Shantsila A, Shantsila E, et al. Atrial fibrillation and hypertension. Hypertension 2017;70(5):854-61.<br><br>Poorer control of hypertension and end organ damage is associated with a higher risk of developing AF
Hyperthyroidism	-	-
Inflammatory bowel disease	-	-
Intensive care unit admission	-	-
Ischaemic heart disease	Chronic	Huxley RR, Lopez FL, Folsom AR, et al. Absolute and attributable risks of atrial fibrillation in relation to optimal and borderline risk factors: the Atherosclerosis Risk in Communities (ARIC) study. Circulation 2011;123(14):1501-08.<br><br>Pizzetti F, Turazza F, Franzosi M, et al. Incidence and prognostic significance of atrial fibrillation in acute myocardial infarction: the GISSI-3 data. Heart 2001;86(5):527-32.<br><br>There is a high risk of AF in the acute setting of myocardial infarction as well as evidence in the context of underlying chronic coronary syndromes.
	Myocardial infarction	
	Percutaneous coronary intervention	
Infection	Gastrointestinal	See table S4
	Influenza	
	Respiratory	
	Sepsis	
	Urinary	
Left ventricular hypertrophy	-	-
Myocarditis	-	-
Obstructive sleep apnoea	-	-
Pulmonary embolus	-	-
Pericarditis	-	-
Pulmonary hypertension	-	-
Peripheral vascular disease	-	-
Rheumatological condition	Autoimmune connective tissue diseases	Lee E, Choi E-K, Jung J-H, et al. Increased risk of atrial fibrillation in patients with Behçet's disease: a nationwide population-based study. Int J Cardiol 2019;292:106-11.<br><br>Moon I, Choi E-K, Jung J-H, et al. Ankylosing spondylitis: a novel risk factor for atrial fibrillation—a nationwide population-based study. Int J Cardiol 2019;275:77-82.<br><br>Melduni RM, Cooper LT, Gersh BJ, et al. Association of Autoimmune Vasculitis and Incident Atrial Fibrillation: A Population-Based Case-Control Study. Journal of the American Heart Association 2020;9(18):e015977.<br><br>Naaraayan A, Meredith A, Nimkar A, et al. Arrhythmia prevalence among patients with Polymyositis-Dermatomyositis in the United States: an observational study. Heart Rhythm 2021
	Rheumatoid arthritis	
	Spondyloarthropathies	
	Vasculitides	

		Songnan W, Shengma C. GW24-e2483 Catheter ablation of atrial fibrillation in patients with autoimmune rheumatic diseases. Heart 2013;99(Suppl 3):A197-A97.<br><br>Giallafos I, Triposkiadis F, Oikonomou E, et al. Incident atrial fibrillation in systemic sclerosis: the predictive role of B-type natriuretic peptide. Hellenic J Cardiol 2014;55:313-21.<br><br>Pugnet G, Gouya H, Puéchal X, et al. Cardiac involvement in granulomatosis with polyangiitis: a magnetic resonance imaging study of 31 consecutive patients. Rheumatology 2017;56(6):947-56.<br><br>Lindhardsen J, Ahlehoff O, Gislason GH, et al. Risk of atrial fibrillation and stroke in rheumatoid arthritis: Danish nationwide cohort study. BMJ 2012;344<br><br>Each of the subtypes of rheumatological disease are associated with differing risks of development of AF. Here they have been categorised in clinical sub-type.
Smoking	Current	See table S4
	Ex	
Surgery (non-cardiac)	Colorectal	See table S4
	Thoracic	
	Vascular	
Systemic Embolism	-	-
Valvular heart disease	Mitral stenosis / rheumatic valvular disease	See table S4
	Non-mitral valve / other valves	
	Mitral regurgitation	
Vascular dementia	-	-

Supplementary Table 2. Definition of disease categories for causes of deaths

Causes of death	Code
**Cardiovascular disorders**	ICD chapter 'Diseases of the circulatory system' (code range: I00–I99), excluding codes relating to infections or cerebrovascular disease.
**Cerebrovascular disorders**	ICD chapter 'Diseases of the circulatory system' (I60-I69)
**Neoplasms**	ICD chapter 'Neoplasms' (C00–D48).
**Infections**	Infectious and parasitic diseases, respiratory infections, urinary tract infections, and cellulitis, as defined by individual codes as Conrad et al.
**Chronic respiratory diseases**	Individual codes Conrad et al.
**Digestive diseases**	ICD chapter 'Diseases of the digestive system' (K00–K93), excepting selected codes categorized as infections.
**Mental and neurological disorders**	ICD chapter 'Mental and behavioral disorders' (F00–F99) and ICD chapter 'Diseases of the nervous system' (G00–G99)
**Injuries**	ICD chapters 'Injury, poisoning and certain other consequences of external causes' (S00–T98) and 'External causes of morbidity and mortality' (V01–Y98)
**Kidney diseases**	ICD sub-chapters 'Renal failure' (N17-N19), 'Glomerular diseases' (N00-N08), 'Renal tubulo-interstitial diseases' (N10-N16), 'Other disorders of kidney and ureter' (N25-N29)

To categorise cause of death as infections or chronic respiratory diseases we used the same codelists as Conrad N, Judge A, Canoy D, et al. Temporal trends and patterns in mortality after incident heart failure: a longitudinal analysis of 86 000 individuals. *JAMA cardiology* 2019;4(11):1102-11

Supplementary Table 3. Read codes and ICD-10 codes used to define the outcomes

Code type	Code	Description
Aortic Stenosis		
Readcode	G541500	Aortic stenosis
Readcode	G541300	Aortic stenosis alone, cause unspecified
Readcode	G541100	Aortic stenosis, non-rheumatic
Readcode	G541400	Aortic valve stenosis with insufficiency
Readcode	G120.00	Rheumatic aortic stenosis
Readcode	G122.00	Rheumatic aortic stenosis with insufficiency
ICD-10	I060	Rheumatic aortic stenosis
ICD-10	I062	Rheumatic aortic stenosis with insufficiency
ICD-10	I350	Nonrheumatic aortic (valve) stenosis
ICD-10	I352	Nonrheumatic aortic (valve) stenosis with insufficiency
Chronic Obstructive Pulmonary Disease		
Readcode	14B3.11	H/O: bronchitis
Readcode	14OJ.00	At risk of chronic obstructive pulmonary disease
Readcode	1J71.00	Suspected chronic obstructive pulmonary disease
Readcode	66Yg.00	Chronic obstructive pulmonary disease disturbs sleep
Readcode	679V.00	Health education - chronic obstructive pulmonary disease
Readcode	8CE6.00	Chronic obstructive pulmonary disease leaflet given
Readcode	H060000	Acute fibrinous bronchitis
Readcode	H060200	Acute pseudomembranous bronchitis
Readcode	H060v00	Subacute bronchitis unspecified
Readcode	H06z200	Recurrent chest infection
Readcode	H30..12	Recurrent wheezy bronchitis
Readcode	H300.00	Tracheobronchitis NOS
Readcode	H301.00	Laryngotracheobronchitis
Readcode	H302.00	Wheezy bronchitis
Readcode	H30z.00	Bronchitis NOS
Readcode	H310.00	Simple chronic bronchitis
Readcode	H310000	Chronic catarrhal bronchitis
Readcode	H310z00	Simple chronic bronchitis NOS
Readcode	H311100	Fetid chronic bronchitis
Readcode	H311z00	Mucopurulent chronic bronchitis NOS
Readcode	H312.00	Obstructive chronic bronchitis
Readcode	H312000	Chronic asthmatic bronchitis
Readcode	H312011	Chronic wheezy bronchitis
Readcode	H313.00	Mixed simple and mucopurulent chronic bronchitis
Readcode	H31y.00	Other chronic bronchitis
Readcode	H31y100	Chronic tracheobronchitis
Readcode	H31yz00	Other chronic bronchitis NOS
Readcode	H32y000	Acute vesicular emphysema
Readcode	H32y100	Atrophic (senile) emphysema
Readcode	H32y111	Acute interstitial emphysema
Readcode	H32y200	MacLeod's unilateral emphysema
Readcode	H3y..00	Other specified chronic obstructive airways disease
Readcode	H581.00	Interstitial emphysema
Readcode	H582.00	Compensatory emphysema
Readcode	66YB.00	Chronic obstructive pulmonary disease monitoring
Readcode	66YD.00	Chronic obstructive pulmonary disease monitoring due
Readcode	66YL.00	Chronic obstructive pulmonary disease follow-up
Readcode	66YM.00	Chronic obstructive pulmonary disease annual review
Readcode	66YS.00	Chronic obstructive pulmonary disease monitoring by nurse
Readcode	66YT.00	Chronic obstructive pulmonary disease monitoring by doctor
Readcode	9Oi..00	Chronic obstructive pulmonary disease monitoring admin
Readcode	9Oi0.00	Chronic obstructive pulmonary disease monitoring 1st letter

Readcode	9Oi1.00	Chronic obstructive pulmonary disease monitoring 2nd letter
Readcode	9Oi2.00	Chronic obstructive pulmonary disease monitoring 3rd letter
Readcode	H3...00	Chronic obstructive pulmonary disease
Readcode	H3...11	Chronic obstructive airways disease
Readcode	H31..00	Chronic bronchitis
Readcode	H312100	Emphysematous bronchitis
Readcode	H312z00	Obstructive chronic bronchitis NOS
Readcode	H31z.00	Chronic bronchitis NOS
Readcode	H32..00	Emphysema
Readcode	H320.00	Chronic bullous emphysema
Readcode	H320000	Segmental bullous emphysema
Readcode	H320100	Zonal bullous emphysema
Readcode	H320200	Giant bullous emphysema
Readcode	H320z00	Chronic bullous emphysema NOS
Readcode	H321.00	Panlobular emphysema
Readcode	H322.00	Centrilobular emphysema
Readcode	H32y.00	Other emphysema
Readcode	H32yz00	Other emphysema NOS
Readcode	H32z.00	Emphysema NOS
Readcode	H36..00	Mild chronic obstructive pulmonary disease
Readcode	H37..00	Moderate chronic obstructive pulmonary disease
Readcode	H38..00	Severe chronic obstructive pulmonary disease
Readcode	H39..00	Very severe chronic obstructive pulmonary disease
Readcode	H3y..11	Other specified chronic obstructive pulmonary disease
Readcode	H3z..00	Chronic obstructive airways disease NOS
Readcode	H3z..11	Chronic obstructive pulmonary disease NOS
Readcode	Hyu3000	[X]Other emphysema
Readcode	Hyu3100	[X]Other specified chronic obstructive pulmonary disease
Readcode	66Yf.00	Number of COPD exacerbations in past year
Readcode	H06..00	Acute bronchitis and bronchiolitis
Readcode	H060.00	Acute bronchitis
Readcode	H060.11	Acute wheezy bronchitis
Readcode	H060300	Acute purulent bronchitis
Readcode	H060400	Acute croupous bronchitis
Readcode	H060500	Acute tracheobronchitis
Readcode	H060600	Acute pneumococcal bronchitis
Readcode	H060700	Acute streptococcal bronchitis
Readcode	H060800	Acute haemophilus influenzae bronchitis
Readcode	H060900	Acute neisseria catarrhalis bronchitis
Readcode	H060A00	Acute bronchitis due to mycoplasma pneumoniae
Readcode	H060B00	Acute bronchitis due to coxsackievirus
Readcode	H060C00	Acute bronchitis due to parainfluenza virus
Readcode	H060D00	Acute bronchitis due to respiratory syncytial virus
Readcode	H060E00	Acute bronchitis due to rhinovirus
Readcode	H060F00	Acute bronchitis due to echovirus
Readcode	H060w00	Acute viral bronchitis unspecified
Readcode	H060x00	Acute bacterial bronchitis unspecified
Readcode	H060z00	Acute bronchitis NOS
Readcode	H06z.00	Acute bronchitis or bronchiolitis NOS
Readcode	H06z000	Chest infection NOS
Readcode	H06z011	Chest infection
Readcode	H20..11	Chest infection - viral pneumonia
Readcode	H21..11	Chest infection - pneumococcal pneumonia
Readcode	H22..11	Chest infection - other bacterial pneumonia
Readcode	H23..11	Chest infection - pneumonia organism OS
Readcode	H24..11	Chest infection with infectious disease EC
Readcode	H25..11	Chest infection - unspecified bronchopneumonia
Readcode	H26..11	Chest infection - pnemonia due to unspecified organism

Wu J, *et al. Open Heart* 2023; 10:e002357. doi: 10.1136/openhrt-2023-002357

Readcode	H270.11	Chest infection - influenza with pneumonia
Readcode	H30..00	Bronchitis unspecified
Readcode	H30..11	Chest infection - unspecified bronchitis
Readcode	H311.00	Mucopurulent chronic bronchitis
Readcode	H311000	Purulent chronic bronchitis
Readcode	Hyu1000	[X]Acute bronchitis due to other specified organisms
Readcode	H312200	Acute exacerbation of chronic obstructive airways disease
Readcode	H3y1.00	Chron obstruct pulmonary dis wth acute exacerbation, unspec
Readcode	8CR1.00	Chronic obstructive pulmonary disease clini management plan
Readcode	9Oi3.00	Chronic obstructive pulmonary disease monitoring verb invite
Readcode	9Oi4.00	Chronic obstructive pulmonary disease monitor phone invite
ICD-10	J40	Bronchitis, not specified as acute or chronic
ICD-10	J41	Simple and mucopurulent chronic bronchitis
ICD-10	J42	Unspecified chronic bronchitis
ICD-10	J43	Emphysema
ICD-10	J448	Other specified chronic obstructive pulmonary disease
ICD-10	J449	Chronic obstructive pulmonary disease, unspecified
ICD-10	J20	Acute bronchitis
ICD-10	J440	Chronic obstructive pulmonary disease with acute lower respiratory infection
ICD-10	J441	Chronic obstructive pulmonary disease with acute exacerbation, unspecified
Chronic Kidney Disease		
Readcode	14D..11	H/O: kidney disease
Readcode	14D..12	H/O: renal disease
Readcode	14D1.00	H/O: nephritis
Readcode	14V2.00	H/O: renal dialysis
Readcode	14V2.11	H/O: kidney dialysis
Readcode	1Z10.00	Chronic kidney disease stage 1
Readcode	1Z11.00	Chronic kidney disease stage 2
Readcode	1Z17.00	Chronic kidney disease stage 1 with proteinuria
Readcode	1Z18.00	Chronic kidney disease stage 1 without proteinuria
Readcode	1Z19.00	Chronic kidney disease stage 2 with proteinuria
Readcode	1Z1A.00	Chronic kidney disease stage 2 without proteinuria
Readcode	A844100	Plasmodium malariae malaria with nephropathy
Readcode	K00..00	Acute glomerulonephritis
Readcode	K00..11	Acute nephritis
Readcode	K000.00	Acute proliferative glomerulonephritis
Readcode	K001.00	Acute nephritis with lesions of necrotising glomerulitis
Readcode	K00y.00	Other acute glomerulonephritis
Readcode	K00y000	Acute glomerulonephritis in diseases EC
Readcode	K00y100	Acute exudative nephritis
Readcode	K00y200	Acute focal nephritis
Readcode	K00y300	Acute diffuse nephritis
Readcode	K00yz00	Other acute glomerulonephritis NOS
Readcode	K00z.00	Acute glomerulonephritis NOS
Readcode	K03T.00	Tubulo-interstit nephritis, not specif as acute or chron
Readcode	K04..00	Acute renal failure
Readcode	K040.00	Acute renal tubular necrosis
Readcode	K041.00	Acute renal cortical necrosis
Readcode	K042.00	Acute renal medullary necrosis
Readcode	K043.00	Acute drug-induced renal failure
Readcode	K044.00	Acute renal failure due to urinary obstruction
Readcode	K04y.00	Other acute renal failure
Readcode	K04z.00	Acute renal failure NOS
Readcode	K08y500	Acute interstitial nephritis
Readcode	K0A0.00	Acute nephritic syndrome
Readcode	K0A0100	Acute nephritic syndrome, focal+segmental glomerular lesions
Readcode	K0A0200	Acute nephritic syn, diffuse membranous glomerulonephritis
Readcode	K0A0500	Acute neph syn, diffuse mesangiocapillary glomerulonephritis

Wu J, *et al. Open Heart* 2023; 10:e002357. doi: 10.1136/openhrt-2023-002357

Readcode	K0A0700	Acute nephrotic syndrm diffuse crescentic glomerulonephritis
Readcode	K101.00	Acute pyelonephritis
Readcode	K101000	Acute pyelonephritis without medullary necrosis
Readcode	K101z00	Acute pyelonephritis NOS
Readcode	Kyu2000	[X]Other acute renal failure
Readcode	L393.00	Acute renal failure following labour and delivery
Readcode	L393000	Post-delivery acute renal failure unspecified
Readcode	L393100	Post-delivery acute renal failure - delivered with p/n prob
Readcode	L393200	Post-delivery acute renal failure with postnatal problem
Readcode	SK05.00	Renal failure following crush syndrome
Readcode	SK05.11	Renal failure after crushing
Readcode	SK08.00	Acute renal failure due to rhabdomyolysis
Readcode	SP15412	Post operative renal failure
Readcode	1Z1..00	Chronic renal impairment
Readcode	1Z12.00	Chronic kidney disease stage 3
Readcode	1Z13.00	Chronic kidney disease stage 4
Readcode	1Z14.00	Chronic kidney disease stage 5
Readcode	1Z15.00	Chronic kidney disease stage 3A
Readcode	1Z16.00	Chronic kidney disease stage 3B
Readcode	1Z1B.00	Chronic kidney disease stage 3 with proteinuria
Readcode	1Z1C.00	Chronic kidney disease stage 3 without proteinuria
Readcode	1Z1D.00	Chronic kidney disease stage 3A with proteinuria
Readcode	1Z1E.00	Chronic kidney disease stage 3A without proteinuria
Readcode	1Z1F.00	Chronic kidney disease stage 3B with proteinuria
Readcode	1Z1G.00	Chronic kidney disease stage 3B without proteinuria
Readcode	1Z1H.00	Chronic kidney disease stage 4 with proteinuria
Readcode	1Z1J.00	Chronic kidney disease stage 4 without proteinuria
Readcode	1Z1K.00	Chronic kidney disease stage 5 with proteinuria
Readcode	1Z1L.00	Chronic kidney disease stage 5 without proteinuria
Readcode	7A60600	Creation of graft fistula for dialysis
Readcode	7B0F100	Pre-transplantation of kidney work-up, recipient
Readcode	7L1A.00	Compensation for renal failure
Readcode	7L1Ay00	Other specified compensation for renal failure
Readcode	7L1Az00	Compensation for renal failure NOS
Readcode	7L1B.00	Placement ambulatory apparatus compensation renal failure
Readcode	7L1By00	Placement ambulatory apparatus- compensate renal failure OS
Readcode	7L1C.00	Placement other apparatus for compensation for renal failure
Readcode	7L1Cz00	Placement other apparatus- compensate for renal failure NOS
Readcode	8L50.00	Renal transplant planned
Readcode	A160000	Tuberculous nephropathy
Readcode	A160200	Tuberculous pyelonephritis
Readcode	A786.00	Haemorrhagic nephrosonephritis
Readcode	C104.11	Diabetic nephropathy
Readcode	C104z00	Diabetes mellitis with nephropathy NOS
Readcode	C108D00	Insulin dependent diabetes mellitus with nephropathy
Readcode	C108D11	Type I diabetes mellitus with nephropathy
Readcode	C109C00	Non-insulin dependent diabetes mellitus with nephropathy
Readcode	C109C11	Type II diabetes mellitus with nephropathy
Readcode	C109C12	Type 2 diabetes mellitus with nephropathy
Readcode	C10ED00	Type 1 diabetes mellitus with nephropathy
Readcode	C10FC00	Type 2 diabetes mellitus with nephropathy
Readcode	C341.00	Gouty nephropathy
Readcode	C341z00	Gouty nephropathy NOS
Readcode	C345.00	Gout due to impairment of renal function
Readcode	C373600	Nephropathic amyloidosis
Readcode	D111300	Haemolytic-uraemic syndrome
Readcode	D215.00	Anaemia secondary to renal failure
Readcode	D215000	Anaemia secondary to chronic renal failure

Readcode	D310100	Henoch-Schonlein nephritis
Readcode	F374A00	Polyneuropathy in uraemia
Readcode	G22..00	Hypertensive renal disease
Readcode	G220.00	Malignant hypertensive renal disease
Readcode	G221.00	Benign hypertensive renal disease
Readcode	G222.00	Hypertensive renal disease with renal failure
Readcode	G22z.00	Hypertensive renal disease NOS
Readcode	G23..00	Hypertensive heart and renal disease
Readcode	G230.00	Malignant hypertensive heart and renal disease
Readcode	G231.00	Benign hypertensive heart and renal disease
Readcode	G232.00	Hypertensive heart&renal dis wth (congestive) heart failure
Readcode	G233.00	Hypertensive heart and renal disease with renal failure
Readcode	G23z.00	Hypertensive heart and renal disease NOS
Readcode	G500400	Acute pericarditis - uraemic
Readcode	Gyu2100	[X]Hypertension secondary to other renal disorders
Readcode	K0...00	Nephritis, nephrosis and nephrotic syndrome
Readcode	K01..00	Nephrotic syndrome
Readcode	K010.00	Nephrotic syndrome with proliferative glomerulonephritis
Readcode	K011.00	Nephrotic syndrome with membranous glomerulonephritis
Readcode	K013.00	Nephrotic syndrome with minimal change glomerulonephritis
Readcode	K013.12	Steroid sensitive nephrotic syndrome
Readcode	K014.00	Nephrotic syndrome, minor glomerular abnormality
Readcode	K015.00	Nephrotic syndrome, focal and segmental glomerular lesions
Readcode	K016.00	Nephrotic syndrome, diffuse membranous glomerulonephritis
Readcode	K017.00	Nephrotic syn difus mesangial prolifertiv glomerulonephritis
Readcode	K018.00	Nephrotic syn,difus endocapilary proliftv glomerulonephritis
Readcode	K019.00	Nephrotic syn,diffuse mesangiocapillary glomerulonephritis
Readcode	K01A.00	Nephrotic syndrome, dense deposit disease
Readcode	K01B.00	Nephrotic syndrome, diffuse crescentic glomerulonephritis
Readcode	K01w.00	Congenital nephrotic syndrome
Readcode	K01x000	Nephrotic syndrome in amyloidosis
Readcode	K01x100	Nephrotic syndrome in diabetes mellitus
Readcode	K01x300	Nephrotic syndrome in polyarteritis nodosa
Readcode	K01x400	Nephrotic syndrome in systemic lupus erythematosus
Readcode	K01x411	Lupus nephritis
Readcode	K01y.00	Nephrotic syndrome with other pathological kidney lesions
Readcode	K01z.00	Nephrotic syndrome NOS
Readcode	K02..00	Chronic glomerulonephritis
Readcode	K02..11	Nephritis - chronic
Readcode	K02..12	Nephropathy - chronic
Readcode	K020.00	Chronic proliferative glomerulonephritis
Readcode	K021.00	Chronic membranous glomerulonephritis
Readcode	K022.00	Chronic membranoproliferative glomerulonephritis
Readcode	K023.00	Chronic rapidly progressive glomerulonephritis
Readcode	K02y.00	Other chronic glomerulonephritis
Readcode	K02y000	Chronic glomerulonephritis + diseases EC
Readcode	K02y200	Chronic focal glomerulonephritis
Readcode	K02y300	Chronic diffuse glomerulonephritis
Readcode	K02yz00	Other chronic glomerulonephritis NOS
Readcode	K02z.00	Chronic glomerulonephritis NOS
Readcode	K03..00	Nephritis and nephropathy unspecified
Readcode	K03..11	Nephritis and nephropathy unspecified
Readcode	K03..12	Nephropathy, unspecified
Readcode	K030.00	Proliferative nephritis unspecified
Readcode	K031.00	Membranous nephritis unspecified
Readcode	K032.00	Membranoproliferative nephritis unspecified
Readcode	K032000	Focal membranoproliferative glomerulonephritis
Readcode	K032300	Anaphylactoid glomerulonephritis

Readcode	K032400	Familial glomerulonephritis in Alport's syndrome
Readcode	K032500	Other familial glomerulonephritis
Readcode	K032600	Berger's IgA or IgG nephropathy
Readcode	K032y00	Nephritis unsp+OS membranoprolif glomerulonephritis lesion
Readcode	K032y11	Hypocomplementaemic persistent glomerulonephritis NEC
Readcode	K032y13	Mesangioproliferative glomerulonephritis NEC
Readcode	K032y14	Mesangiocapillary glomerulonephritis NEC
Readcode	K032y15	Mixed membranous and proliferative glomerulonephritis NEC
Readcode	K032z00	Nephritis unsp+membranoprolif glomerulonephritis lesion NOS
Readcode	K033.00	Rapidly progressive nephritis unspecified
Readcode	K034.00	Renal cortical necrosis unspecified
Readcode	K035.00	Renal medullary necrosis unspecified
Readcode	K03U.00	Unspecif nephr synd, diff concentric glomerulonephritis
Readcode	K03V.00	Unspecified nephritic syndrome, dense deposit disease
Readcode	K03W.00	Unsp nephrit synd, diff endocap prolif glomerulonephritis
Readcode	K03X.00	Unsp nephrit synd, diff mesang prolif glomerulonephritis
Readcode	K03y.00	Other nephritis and nephrosis unspecified
Readcode	K03y000	Other nephritis and nephrosis in diseases EC
Readcode	K03y200	Other interstitial nephritis
Readcode	K03yz00	Other nephritis and nephrosis NOS
Readcode	K03z.00	Unspecified glomerulonephritis NOS
Readcode	K05..00	Chronic renal failure
Readcode	K05..11	Chronic uraemia
Readcode	K05..12	End stage renal failure
Readcode	K050.00	End stage renal failure
Readcode	K06..00	Renal failure unspecified
Readcode	K06..11	Uraemia NOS
Readcode	K060.00	Renal impairment
Readcode	K060.11	Impaired renal function
Readcode	K08..00	Impaired renal function disorder
Readcode	K080.00	Renal osteodystrophy
Readcode	K080000	Phosphate-losing tubular disorders
Readcode	K080100	Renal dwarfism
Readcode	K080200	Renal infantilism
Readcode	K080300	Renal rickets
Readcode	K080z00	Renal osteodystrophy NOS
Readcode	K081.00	Nephrogenic diabetes insipidus
Readcode	K08y.00	Other impaired renal function disorder
Readcode	K08y000	Hypokalaemic nephropathy
Readcode	K08y300	Renal function impairment with growth failure
Readcode	K08y400	Renal tubular acidosis
Readcode	K08yz00	Other impaired renal function disorder NOS
Readcode	K08yz11	Renal acidaemia
Readcode	K08z.00	Impaired renal function disorder NOS
Readcode	K0A0300	Acut neph syn, diffuse mesangial prolifrative glomnephritis
Readcode	K0A1.00	Rapidly progressive nephritic syndrome
Readcode	K0A1100	Rapid progres nephritic syn focal+segmental glomerulr lesion
Readcode	K0A1200	Rapid progres neph syn diffuse membranous glomerulonephritis
Readcode	K0A1300	Rpd prog neph syn df mesangial prolifratv glomerulonephritis
Readcode	K0A1600	Rapid progressive nephritic syndrome, dense deposit disease
Readcode	K0A1700	Rapid progres nephritic syn df crescentic glomerulonephritis
Readcode	K0A2200	Recur+persist haematuria difus membranous glomerulonephritis
Readcode	K0A2300	Recur+persist haemuria df mesangial prolif glomerulnephritis
Readcode	K0A2500	Recur+persist hmuria df mesangiocapilary glomerulonephritis
Readcode	K0A2700	Recur+persist haematuria difus crescentic glomerulonephritis
Readcode	K0A2800	IgA nephropathy
Readcode	K0A3.00	Chronic nephritic syndrome
Readcode	K0A3000	Chronic nephritic syndrome, minor glomerular abnormality

Readcode	K0A3100	Chronic nephritic syndrm focal+segmental glomerular lesions
Readcode	K0A3200	Chron nephritic syndrom difuse membranous glomerulonephritis
Readcode	K0A3300	Chron neph syn difus mesangial prolifrtiv glomerulonephritis
Readcode	K0A3500	Chronic neph syn difus mesangiocapillary glomerulonephritis
Readcode	K0A3600	Chronic nephritic syndrome, dense deposit disease
Readcode	K0A3700	Chronic nephritic syn diffuse crescentic glomerulonephritis
Readcode	K0A4500	Isoltd prteinur+specfd morph les df mesangiocap glomnephr
Readcode	K0A5.00	Hereditary nephropathy not elsewhere classified
Readcode	K0A5000	Hereditary nephropathy NEC, minor glomerular abnormality
Readcode	K0A5100	Hereditary nephropathy NEC,focal+segmnt glomerular lesion
Readcode	K0A5200	Hereditry nephropathy NEC,difus membran glomerulnephritis
Readcode	K0A5300	Heredtry nephrpthy NEC difus mesangial prolif glomnephrit
Readcode	K0A5600	Hereditary nephropathy, NEC, dense deposit disease
Readcode	K0A5X00	Hereditary nephropathy, unspecif morphological changes
Readcode	K0B..00	Renal tubulo-interstitial disorders in diseases EC
Readcode	K0B1.00	Renal tubulo-interstitial disorder/ neoplastic diseases
Readcode	K0B4000	Renal tubulo-interstitial disorder in SLE
Readcode	K0C0.00	Analgesic nephropathy
Readcode	K0C1.00	Nephropathy induced by other drugs meds and biologl substncs
Readcode	K0C2.00	Nephropathy induced by unspec drug medicament or biol subs
Readcode	K0C4.00	Toxic nephropathy, not elsewhere classified
Readcode	K0D..00	End-stage renal disease
Readcode	K0y..00	Other specified nephritis, nephrosis or nephrotic syndrome
Readcode	K0z..00	Nephritis, nephrosis and nephrotic syndrome NOS
Readcode	K100.00	Chronic pyelonephritis
Readcode	K100100	Chronic pyelonephritis with medullary necrosis
Readcode	K100400	Nonobstructive reflux-associated chronic pyelonephritis
Readcode	K100500	Chronic obstructive pyelonephritis
Readcode	K100600	Calculous pyelonephritis
Readcode	K100z00	Chronic pyelonephritis NOS
Readcode	K104.00	Xanthogranulomatous pyelonephritis
Readcode	K10y.00	Pyelonephritis and pyonephrosis unspecified
Readcode	K10y000	Pyelonephritis unspecified
Readcode	K10y300	Pyelonephritis in diseases EC
Readcode	K10yz00	Unspecified pyelonephritis NOS
Readcode	K13..00	Other kidney and ureter disorders
Readcode	K13..11	Other kidney disorders
Readcode	K138.00	Vascular disorders of kidney
Readcode	K138.11	Renal vascular disorders
Readcode	K138z00	Renal vascular disorders NOS
Readcode	K13y.00	Other kidney and ureteric disorders
Readcode	K13yz00	Other kidney and ureteric disorders NOS
Readcode	K13yz11	Salt-losing nephritis
Readcode	K13z.00	Kidney and ureter disease NOS
Readcode	Kyu0900	[X]Unsp nephrit synd, diff mesang prolif glomerulonephritis
Readcode	Kyu1.00	[X]Renal tubulo-interstitial diseases
Readcode	Kyu1400	[X]Nephropathy induced by other drugs+biological substances
Readcode	Kyu2.00	[X]Renal failure
Readcode	Kyu2100	[X]Other chronic renal failure
Readcode	Kyu4.00	[X]Other disorders of kidney and ureter
Readcode	Kyu4000	[X]Other disorders resulting/impaired renal tubular function
Readcode	Kyu4100	[X]Other specified disorders of kidney and ureter
Readcode	L093.00	Renal failure following abortive pregnancy
Readcode	L093400	Uraemia following abortive pregnancy
Readcode	L162.00	Unspecified renal disease in pregnancy
Readcode	L162.12	Nephropathy NOS in pregnancy without hypertension
Readcode	L162.13	Uraemia in pregnancy without hypertension
Readcode	L162000	Unspecified renal disease in pregnancy unspecified

Wu J, *et al. Open Heart* 2023; 10:e002357. doi: 10.1136/openhrt-2023-002357

Readcode	L162100	Unspecified renal disease in pregnancy - delivered
Readcode	PD1..00	Congenital cystic kidney disease
Readcode	PD1..11	Congenital cystic renal disease
Readcode	PD11.00	Polycystic kidney disease
Readcode	PD11z00	Polycystic kidney disease NOS
Readcode	PD11z11	Cystic kidney disease NEC
Readcode	PD1y.00	Other specified congenital cystic kidney disease
Readcode	PD1y000	Fibrocystic kidney disease
Readcode	PD1yz00	Other congenital cystic kidney disease NOS
Readcode	PD1z.00	Congenital cystic kidney disease NOS
Readcode	Pyu7000	[X]Other cystic kidney diseases
Readcode	Q001.00	Fetus or neonate affected by maternal renal/urinary disease
Readcode	Q48y000	Congenital renal failure
Readcode	SP15400	Renal failure as a complication of care
Readcode	SP15411	Kidney failure as a complication of care
Readcode	SP15413	Uraemia - post operative
Readcode	Z1A2.00	Haemodialysis training
Readcode	ZV56100	[V]Preparatory care for dialysis
Readcode	7A61900	Ligation of arteriovenous dialysis fistula
Readcode	7L1A.11	Dialysis for renal failure
Readcode	7L1A000	Renal dialysis
Readcode	7L1A100	Peritoneal dialysis
Readcode	7L1A200	Haemodialysis NEC
Readcode	7L1A400	Automated peritoneal dialysis
Readcode	7L1A500	Continuous ambulatory peritoneal dialysis
Readcode	7L1A600	Peritoneal dialysis NEC
Readcode	7L1B.11	Placement ambulatory dialysis apparatus - compens renal fail
Readcode	7L1B000	Insertion of ambulatory peritoneal dialysis catheter
Readcode	7L1B100	Removal of ambulatory peritoneal dialysis catheter
Readcode	7L1C000	Insertion of temporary peritoneal dialysis catheter
Readcode	SP01500	Mechanical complication of dialysis catheter
Readcode	SP05613	[X] Peritoneal dialysis associated peritonitis
Readcode	SP07G00	Stenosis of arteriovenous dialysis fistula
Readcode	TA02000	Accid cut,puncture,perf,h'ge - kidney dialysis
Readcode	TA22000	Failure of sterile precautions during kidney dialysis
Readcode	TB11.00	Kidney dialysis with complication, without blame
Readcode	TB11.11	Renal dialysis with complication, without blame
Readcode	U612200	[X]Failure sterile precautions dur kidney dialys/other perf
Readcode	Z919.00	Care of haemodialysis equipment
Readcode	Z919100	Priming haemodialysis lines
Readcode	Z919300	Reversing haemodialysis lines
Readcode	Z91A.00	Peritoneal dialysis bag procedure
Readcode	ZV45100	[V]Renal dialysis status
Readcode	ZV56.00	[V]Aftercare involving intermittent dialysis
Readcode	ZV56011	[V]Aftercare involving renal dialysis NOS
Readcode	ZV56y00	[V]Other specified aftercare involving intermittent dialysis
Readcode	ZV56y11	[V]Aftercare involving peritoneal dialysis
Readcode	ZV56z00	[V]Unspecified aftercare involving intermittent dialysis
Readcode	ZVu3G00	[X]Other dialysis
Readcode	7B00.00	Transplantation of kidney
Readcode	7B00000	Autotransplant of kidney
Readcode	7B00100	Transplantation of kidney from live donor
Readcode	7B00111	Allotransplantation of kidney from live donor
Readcode	7B00200	Transplantation of kidney from cadaver
Readcode	7B00211	Allotransplantation of kidney from cadaver
Readcode	7B00300	Allotransplantation of kidney from cadaver, heart-beating
Readcode	7B00400	Allotransplantation kidney from cadaver, heart non-beating
Readcode	7B00y00	Other specified transplantation of kidney

Readcode	7B00z00	Transplantation of kidney NOS
Readcode	7B01500	Transplant nephrectomy
Readcode	7B01511	Excision of rejected transplanted kidney
Readcode	7B06300	Exploration of renal transplant
Readcode	7B0F.00	Interventions associated with transplantation of kidney
Readcode	K0B5.00	Renal tubulo-interstitial disordrs in transplant rejectn
Readcode	SP08300	Kidney transplant failure and rejection
Readcode	TB00100	Kidney transplant with complication, without blame
Readcode	TB00111	Renal transplant with complication, without blame
Readcode	ZV42000	[V]Kidney transplanted
Readcode	66i..00	Chronic kidney disease monitoring
Readcode	6AA..00	Chronic kidney disease annual review
Readcode	9Ot..00	Chronic kidney disease monitoring administration
Readcode	9Ot0.00	Chronic kidney disease monitoring first letter
Readcode	9Ot1.00	Chronic kidney disease monitoring second letter
Readcode	9Ot2.00	Chronic kidney disease monitoring third letter
Readcode	9Ot3.00	Chronic kidney disease monitoring verbal invite
Readcode	9Ot4.00	Chronic kidney disease monitoring telephone invite
Readcode	14D..11	Chronic kidney disease, stage 1
Readcode	14D..12	Chronic kidney disease, stage 2
Readcode	14D1.00	Acute nephritic syndrome
Readcode	14V2.00	Acute tubulo-interstitial nephritis
Readcode	14V2.11	Acute renal failure
Readcode	1Z10.00	Rapidly progressive nephritic syndrome
Readcode	1Z11.00	Chronic nephritic syndrome
Readcode	1Z17.00	Unspecified nephritic syndrome ; Diffuse membranous glomerulonephritis
Readcode	1Z18.00	Unspecified nephritic syndrome ; Diffuse mesangial proliferative glomerulonephritis
Readcode	1Z19.00	Unspecified nephritic syndrome ; Diffuse endocapillary proliferative glomerulonephritis
Readcode	1Z1A.00	Unspecified nephritic syndrome ; Diffuse mesangiocapillary glomerulonephritis
Readcode	A844100	Unspecified nephritic syndrome ; Dense deposit disease
Readcode	K00..00	Hereditary nephropathy, not elsewhere classified ; Diffuse membranous glomerulonephritis
Readcode	K00..11	Hereditary nephropathy, not elsewhere classified ; Diffuse mesangial proliferative glomerulonephritis
Readcode	K000.00	Hereditary nephropathy, not elsewhere classified ; Diffuse endocapillary proliferative glomerulonephritis
Readcode	K001.00	Chronic kidney disease, stage 3
Readcode	K00y.00	Chronic kidney disease, stage 4
Readcode	K00y000	Chronic kidney disease, stage 5
Readcode	K00y100	Chronic kidney disease, unspecified
Readcode	K00y200	Unspecified kidney failure
Readcode	K00y300	Disorders resulting from impaired renal tubular function
Readcode	K00yz00	Kidney dialysis
Readcode	K00z.00	Care involving dialysis
Readcode	K03T.00	Dependence on renal dialysis
Readcode	K04..00	Kidney transplant failure and rejection
Readcode	K040.00	Kidney transplant status
Readcode	K041.00	H/O: kidney disease
Readcode	K042.00	H/O: renal disease
Readcode	K043.00	H/O: nephritis
Readcode	K044.00	H/O: renal dialysis
Readcode	K04y.00	H/O: kidney dialysis
Readcode	K04z.00	Chronic kidney disease stage 1
Readcode	K08y500	Chronic kidney disease stage 2
Readcode	K0A0.00	Chronic kidney disease stage 1 with proteinuria
Readcode	K0A0100	Chronic kidney disease stage 1 without proteinuria

Readcode	K0A0200	Chronic kidney disease stage 2 with proteinuria
Readcode	K0A0500	Chronic kidney disease stage 2 without proteinuria
Readcode	K0A0700	Plasmodium malariae malaria with nephropathy
Readcode	K101.00	Acute glomerulonephritis
Readcode	K101000	Acute nephritis
Readcode	K101z00	Acute proliferative glomerulonephritis
Readcode	Kyu2000	Acute nephritis with lesions of necrotising glomerulitis
Readcode	L393.00	Other acute glomerulonephritis
Readcode	L393000	Acute glomerulonephritis in diseases EC
Readcode	L393100	Acute exudative nephritis
Readcode	L393200	Acute focal nephritis
Readcode	SK05.00	Acute diffuse nephritis
Readcode	SK05.11	Other acute glomerulonephritis NOS
Readcode	SK08.00	Acute glomerulonephritis NOS
Readcode	SP15412	Tubulo-interstit nephritis, not specif as acute or chron
Readcode	1Z1..00	Acute renal failure
Readcode	1Z12.00	Acute renal tubular necrosis
Readcode	1Z13.00	Acute renal cortical necrosis
Readcode	1Z14.00	Acute renal medullary necrosis
Readcode	1Z15.00	Acute drug-induced renal failure
Readcode	1Z16.00	Acute renal failure due to urinary obstruction
Readcode	1Z1B.00	Other acute renal failure
Readcode	1Z1C.00	Acute renal failure NOS
Readcode	1Z1D.00	Acute interstitial nephritis
Readcode	1Z1E.00	Acute nephritic syndrome
Readcode	1Z1F.00	Acute nephritic syndrome, focal+segmental glomerular lesions
Readcode	1Z1G.00	Acute nephritic syn, diffuse membranous glomerulonephritis
Readcode	1Z1H.00	Acute neph syn, diffuse mesangiocapillary glomerulonephritis
Readcode	1Z1J.00	Acute nephrotic syndrm diffuse crescentic glomerulonephritis
Readcode	1Z1K.00	Acute pyelonephritis
Readcode	1Z1L.00	Acute pyelonephritis without medullary necrosis
Readcode	7A60600	Acute pyelonephritis NOS
Readcode	7B0F100	[X]Other acute renal failure
Readcode	7L1A.00	Acute renal failure following labour and delivery
Readcode	7L1Ay00	Post-delivery acute renal failure unspecified
Readcode	7L1Az00	Post-delivery acute renal failure - delivered with p/n prob
Readcode	7L1B.00	Post-delivery acute renal failure with postnatal problem
Readcode	7L1By00	Renal failure following crush syndrome
Readcode	7L1C.00	Renal failure after crushing
Readcode	7L1Cz00	Acute renal failure due to rhabdomyolysis
Readcode	8L50.00	Post operative renal failure
Readcode	A160000	Chronic renal impairment
Readcode	A160200	Chronic kidney disease stage 3
Readcode	A786.00	Chronic kidney disease stage 4
Readcode	C104.11	Chronic kidney disease stage 5
Readcode	C104z00	Chronic kidney disease stage 3A
Readcode	C108D00	Chronic kidney disease stage 3B
Readcode	C108D11	Chronic kidney disease stage 3 with proteinuria
Readcode	C109C00	Chronic kidney disease stage 3 without proteinuria
Readcode	C109C11	Chronic kidney disease stage 3A with proteinuria
Readcode	C109C12	Chronic kidney disease stage 3A without proteinuria
Readcode	C10ED00	Chronic kidney disease stage 3B with proteinuria
Readcode	C10FC00	Chronic kidney disease stage 3B without proteinuria
Readcode	C341.00	Chronic kidney disease stage 4 with proteinuria
Readcode	C341z00	Chronic kidney disease stage 4 without proteinuria
Readcode	C345.00	Chronic kidney disease stage 5 with proteinuria
Readcode	C373600	Chronic kidney disease stage 5 without proteinuria
Readcode	D111300	Creation of graft fistula for dialysis

Readcode	D215.00	Pre-transplantation of kidney work-up, recipient
Readcode	D215000	Compensation for renal failure
Readcode	D310100	Other specified compensation for renal failure
Readcode	F374A00	Compensation for renal failure NOS
Readcode	G22..00	Placement ambulatory apparatus compensation renal failure
Readcode	G220.00	Placement ambulatory apparatus- compensate renal failure OS
Readcode	G221.00	Placement other apparatus for compensation for renal failure
Readcode	G222.00	Placement other apparatus- compensate for renal failure NOS
Readcode	G22z.00	Renal transplant planned
Readcode	G23..00	Tuberculous nephropathy
Readcode	G230.00	Tuberculous pyelonephritis
Readcode	G231.00	Haemorrhagic nephrosonephritis
Readcode	G232.00	Diabetic nephropathy
Readcode	G233.00	Diabetes mellitis with nephropathy NOS
Readcode	G23z.00	Insulin dependent diabetes mellitus with nephropathy
Readcode	G500400	Type I diabetes mellitus with nephropathy
Readcode	Gyu2100	Non-insulin dependent diabetes mellitus with nephropathy
Readcode	K0...00	Type II diabetes mellitus with nephropathy
Readcode	K01..00	Type 2 diabetes mellitus with nephropathy
Readcode	K010.00	Type 1 diabetes mellitus with nephropathy
Readcode	K011.00	Type 2 diabetes mellitus with nephropathy
Readcode	K013.00	Gouty nephropathy
Readcode	K013.12	Gouty nephropathy NOS
Readcode	K014.00	Gout due to impairment of renal function
Readcode	K015.00	Nephropathic amyloidosis
Readcode	K016.00	Haemolytic-uraemic syndrome
Readcode	K017.00	Anaemia secondary to renal failure
Readcode	K018.00	Anaemia secondary to chronic renal failure
Readcode	K019.00	Henoch-Schonlein nephritis
Readcode	K01A.00	Polyneuropathy in uraemia
Readcode	K01B.00	Hypertensive renal disease
Readcode	K01w.00	Malignant hypertensive renal disease
Readcode	K01x000	Benign hypertensive renal disease
Readcode	K01x100	Hypertensive renal disease with renal failure
Readcode	K01x300	Hypertensive renal disease NOS
Readcode	K01x400	Hypertensive heart and renal disease
Readcode	K01x411	Malignant hypertensive heart and renal disease
Readcode	K01y.00	Benign hypertensive heart and renal disease
Readcode	K01z.00	Hypertensive heart&renal dis wth (congestive) heart failure
Readcode	K02..00	Hypertensive heart and renal disease with renal failure
Readcode	K02..11	Hypertensive heart and renal disease NOS
Readcode	K02..12	Acute pericarditis - uraemic
Readcode	K020.00	[X]Hypertension secondary to other renal disorders
Readcode	K021.00	Nephritis, nephrosis and nephrotic syndrome
Readcode	K022.00	Nephrotic syndrome
Readcode	K023.00	Nephrotic syndrome with proliferative glomerulonephritis
Readcode	K02y.00	Nephrotic syndrome with membranous glomerulonephritis
Readcode	K02y000	Nephrotic syndrome with minimal change glomerulonephritis
Readcode	K02y200	Steroid sensitive nephrotic syndrome
Readcode	K02y300	Nephrotic syndrome, minor glomerular abnormality
Readcode	K02yz00	Nephrotic syndrome, focal and segmental glomerular lesions
Readcode	K02z.00	Nephrotic syndrome, diffuse membranous glomerulonephritis
Readcode	K03..00	Nephrotic syn difus mesangial prolifertiv glomerulonephritis
Readcode	K03..11	Nephrotic syn,difus endocapilary proliftv glomerulonephritis
Readcode	K03..12	Nephrotic syn,diffuse mesangiocapillary glomerulonephritis
Readcode	K030.00	Nephrotic syndrome, dense deposit disease
Readcode	K031.00	Nephrotic syndrome, diffuse crescentic glomerulonephritis
Readcode	K032.00	Congenital nephrotic syndrome

Readcode	K032000	Nephrotic syndrome in amyloidosis
Readcode	K032300	Nephrotic syndrome in diabetes mellitus
Readcode	K032400	Nephrotic syndrome in polyarteritis nodosa
Readcode	K032500	Nephrotic syndrome in systemic lupus erythematosus
Readcode	K032600	Lupus nephritis
Readcode	K032y00	Nephrotic syndrome with other pathological kidney lesions
Readcode	K032y11	Nephrotic syndrome NOS
Readcode	K032y13	Chronic glomerulonephritis
Readcode	K032y14	Nephritis - chronic
Readcode	K032y15	Nephropathy - chronic
Readcode	K032z00	Chronic proliferative glomerulonephritis
Readcode	K033.00	Chronic membranous glomerulonephritis
Readcode	K034.00	Chronic membranoproliferative glomerulonephritis
Readcode	K035.00	Chronic rapidly progressive glomerulonephritis
Readcode	K03U.00	Other chronic glomerulonephritis
Readcode	K03V.00	Chronic glomerulonephritis + diseases EC
Readcode	K03W.00	Chronic focal glomerulonephritis
Readcode	K03X.00	Chronic diffuse glomerulonephritis
Readcode	K03y.00	Other chronic glomerulonephritis NOS
Readcode	K03y000	Chronic glomerulonephritis NOS
Readcode	K03y200	Nephritis and nephropathy unspecified
Readcode	K03yz00	Nephritis and nephropathy unspecified
Readcode	K03z.00	Nephropathy, unspecified
Readcode	K05..00	Proliferative nephritis unspecified
Readcode	K05..11	Membranous nephritis unspecified
Readcode	K05..12	Membranoproliferative nephritis unspecified
Readcode	K050.00	Focal membranoproliferative glomerulonephritis
Readcode	K06..00	Anaphylactoid glomerulonephritis
Readcode	K06..11	Familial glomerulonephritis in Alport's syndrome
Readcode	K060.00	Other familial glomerulonephritis
Readcode	K060.11	Berger's IgA or IgG nephropathy
Readcode	K08..00	Nephritis unsp+OS membranoprolif glomerulonephritis lesion
Readcode	K080.00	Hypocomplementaemic persistent glomerulonephritis NEC
Readcode	K080000	Mesangioproliferative glomerulonephritis NEC
Readcode	K080100	Mesangiocapillary glomerulonephritis NEC
Readcode	K080200	Mixed membranous and proliferative glomerulonephritis NEC
Readcode	K080300	Nephritis unsp+membranoprolif glomerulonephritis lesion NOS
Readcode	K080z00	Rapidly progressive nephritis unspecified
Readcode	K081.00	Renal cortical necrosis unspecified
Readcode	K08y.00	Renal medullary necrosis unspecified
Readcode	K08y000	Unspecif nephr synd, diff concentric glomerulonephritis
Readcode	K08y300	Unspecified nephritic syndrome, dense deposit disease
Readcode	K08y400	Unsp nephrit synd, diff endocap prolif glomerulonephritis
Readcode	K08yz00	Unsp nephrit synd, diff mesang prolif glomerulonephritis
Readcode	K08yz11	Other nephritis and nephrosis unspecified
Readcode	K08z.00	Other nephritis and nephrosis in diseases EC
Readcode	K0A0300	Other interstitial nephritis
Readcode	K0A1.00	Other nephritis and nephrosis NOS
Readcode	K0A1100	Unspecified glomerulonephritis NOS
Readcode	K0A1200	Chronic renal failure
Readcode	K0A1300	Chronic uraemia
Readcode	K0A1600	End stage renal failure
Readcode	K0A1700	End stage renal failure
Readcode	K0A2200	Renal failure unspecified
Readcode	K0A2300	Uraemia NOS
Readcode	K0A2500	Renal impairment
Readcode	K0A2700	Impaired renal function
Readcode	K0A2800	Impaired renal function disorder

Readcode	K0A3.00	Renal osteodystrophy
Readcode	K0A3000	Phosphate-losing tubular disorders
Readcode	K0A3100	Renal dwarfism
Readcode	K0A3200	Renal infantilism
Readcode	K0A3300	Renal rickets
Readcode	K0A3500	Renal osteodystrophy NOS
Readcode	K0A3600	Nephrogenic diabetes insipidus
Readcode	K0A3700	Other impaired renal function disorder
Readcode	K0A4500	Hypokalaemic nephropathy
Readcode	K0A5.00	Renal function impairment with growth failure
Readcode	K0A5000	Renal tubular acidosis
Readcode	K0A5100	Other impaired renal function disorder NOS
Readcode	K0A5200	Renal acidaemia
Readcode	K0A5300	Impaired renal function disorder NOS
Readcode	K0A5600	Acut neph syn, diffuse mesangial prolifrative glomnephritis
Readcode	K0A5X00	Rapidly progressive nephritic syndrome
Readcode	K0B..00	Rapid progres nephritic syn focal+segmental glomerulr lesion
Readcode	K0B1.00	Rapid progres neph syn diffuse membranous glomerulonephritis
Readcode	K0B4000	Rpd prog neph syn df mesangial prolifratv glomerulonephritis
Readcode	K0C0.00	Rapid progressive nephritic syndrome, dense deposit disease
Readcode	K0C1.00	Rapid progres nephritic syn df crescentic glomerulonephritis
Readcode	K0C2.00	Recur+persist haematuria difus membranous glomerulonephritis
Readcode	K0C4.00	Recur+persist haemuria df mesangial prolif glomerulnephritis
Readcode	K0D..00	Recur+persist hmuria df mesangiocapilary glomerulonephritis
Readcode	K0y..00	Recur+persist haematuria difus crescentic glomerulonephritis
Readcode	K0z..00	IgA nephropathy
Readcode	K100.00	Chronic nephritic syndrome
Readcode	K100100	Chronic nephritic syndrome, minor glomerular abnormality
Readcode	K100400	Chronic nephritic syndrm focal+segmental glomerular lesions
Readcode	K100500	Chron nephritic syndrom difuse membranous glomerulonephritis
Readcode	K100600	Chron neph syn difus mesangial prolifrtiv glomerulonephritis
Readcode	K100z00	Chronic neph syn difus mesangiocapillary glomerulonephritis
Readcode	K104.00	Chronic nephritic syndrome, dense deposit disease
Readcode	K10y.00	Chronic nephritic syn diffuse crescentic glomerulonephritis
Readcode	K10y000	Isoltd prteinur+specfd morph les df mesangiocap glomnephr
Readcode	K10y300	Hereditary nephropathy not elsewhere classified
Readcode	K10yz00	Hereditary nephropathy NEC, minor glomerular abnormality
Readcode	K13..00	Hereditary nephropathy NEC,focal+segmnt glomerular lesion
Readcode	K13..11	Hereditry nephropathy NEC,difus membran glomerulnephritis
Readcode	K138.00	Heredtry nephrpthy NEC difus mesangial prolif glomnephrit
Readcode	K138.11	Hereditary nephropathy, NEC, dense deposit disease
Readcode	K138z00	Hereditary nephropathy, unspecif morphological changes
Readcode	K13y.00	Renal tubulo-interstitial disorders in diseases EC
Readcode	K13yz00	Renal tubulo-interstitial disorder/ neoplastic diseases
Readcode	K13yz11	Renal tubulo-interstitial disorder in SLE
Readcode	K13z.00	Analgesic nephropathy
Readcode	Kyu0900	Nephropathy induced by other drugs meds and biologl substncs
Readcode	Kyu1.00	Nephropathy induced by unspec drug medicament or biol subs
Readcode	Kyu1400	Toxic nephropathy, not elsewhere classified
Readcode	Kyu2.00	End-stage renal disease
Readcode	Kyu2100	Other specified nephritis, nephrosis or nephrotic syndrome
Readcode	Kyu4.00	Nephritis, nephrosis and nephrotic syndrome NOS
Readcode	Kyu4000	Chronic pyelonephritis
Readcode	Kyu4100	Chronic pyelonephritis with medullary necrosis
Readcode	L093.00	Nonobstructive reflux-associated chronic pyelonephritis
Readcode	L093400	Chronic obstructive pyelonephritis
Readcode	L162.00	Calculous pyelonephritis
Readcode	L162.12	Chronic pyelonephritis NOS

Readcode	L162.13	Xanthogranulomatous pyelonephritis
Readcode	L162000	Pyelonephritis and pyonephrosis unspecified
Readcode	L162100	Pyelonephritis unspecified
Readcode	PD1..00	Pyelonephritis in diseases EC
Readcode	PD1..11	Unspecified pyelonephritis NOS
Readcode	PD11.00	Other kidney and ureter disorders
Readcode	PD11z00	Other kidney disorders
Readcode	PD11z11	Vascular disorders of kidney
Readcode	PD1y.00	Renal vascular disorders
Readcode	PD1y000	Renal vascular disorders NOS
Readcode	PD1yz00	Other kidney and ureteric disorders
Readcode	PD1z.00	Other kidney and ureteric disorders NOS
Readcode	Pyu7000	Salt-losing nephritis
Readcode	Q001.00	Kidney and ureter disease NOS
Readcode	Q48y000	[X]Unsp nephrit synd, diff mesang prolif glomerulonephritis
Readcode	SP15400	[X]Renal tubulo-interstitial diseases
Readcode	SP15411	[X]Nephropathy induced by other drugs+biological substances
Readcode	SP15413	[X]Renal failure
Readcode	Z1A2.00	[X]Other chronic renal failure
Readcode	ZV56100	[X]Other disorders of kidney and ureter
Readcode	7A61900	[X]Other disorders resulting/impaired renal tubular function
Readcode	7L1A.11	[X]Other specified disorders of kidney and ureter
Readcode	7L1A000	Renal failure following abortive pregnancy
Readcode	7L1A100	Uraemia following abortive pregnancy
Readcode	7L1A200	Unspecified renal disease in pregnancy
Readcode	7L1A400	Nephropathy NOS in pregnancy without hypertension
Readcode	7L1A500	Uraemia in pregnancy without hypertension
Readcode	7L1A600	Unspecified renal disease in pregnancy unspecified
Readcode	7L1B.11	Unspecified renal disease in pregnancy - delivered
Readcode	7L1B000	Congenital cystic kidney disease
Readcode	7L1B100	Congenital cystic renal disease
Readcode	7L1C000	Polycystic kidney disease
Readcode	SP01500	Polycystic kidney disease NOS
Readcode	SP05613	Cystic kidney disease NEC
Readcode	SP07G00	Other specified congenital cystic kidney disease
Readcode	TA02000	Fibrocystic kidney disease
Readcode	TA22000	Other congenital cystic kidney disease NOS
Readcode	TB11.00	Congenital cystic kidney disease NOS
Readcode	TB11.11	[X]Other cystic kidney diseases
Readcode	U612200	Fetus or neonate affected by maternal renal/urinary disease
Readcode	Z919.00	Congenital renal failure
Readcode	Z919100	Renal failure as a complication of care
Readcode	Z919300	Kidney failure as a complication of care
Readcode	Z91A.00	Uraemia - post operative
Readcode	ZV45100	Haemodialysis training
Readcode	ZV56.00	[V]Preparatory care for dialysis
Readcode	ZV56011	Ligation of arteriovenous dialysis fistula
Readcode	ZV56y00	Dialysis for renal failure
Readcode	ZV56y11	Renal dialysis
Readcode	ZV56z00	Peritoneal dialysis
Readcode	ZVu3G00	Haemodialysis NEC
Readcode	7B00.00	Automated peritoneal dialysis
Readcode	7B00000	Continuous ambulatory peritoneal dialysis
Readcode	7B00100	Peritoneal dialysis NEC
Readcode	7B00111	Placement ambulatory dialysis apparatus - compens renal fail
Readcode	7B00200	Insertion of ambulatory peritoneal dialysis catheter
Readcode	7B00211	Removal of ambulatory peritoneal dialysis catheter
Readcode	7B00300	Insertion of temporary peritoneal dialysis catheter

Readcode	7B00400	Mechanical complication of dialysis catheter
Readcode	7B00y00	[X] Peritoneal dialysis associated peritonitis
Readcode	7B00z00	Stenosis of arteriovenous dialysis fistula
Readcode	7B01500	Accid cut,puncture,perf,h'ge - kidney dialysis
Readcode	7B01511	Failure of sterile precautions during kidney dialysis
Readcode	7B06300	Kidney dialysis with complication, without blame
Readcode	7B0F.00	Renal dialysis with complication, without blame
Readcode	K0B5.00	[X]Failure sterile precautions dur kidney dialys/other perf
Readcode	SP08300	Care of haemodialysis equipment
Readcode	TB00100	Priming haemodialysis lines
Readcode	TB00111	Reversing haemodialysis lines
Readcode	ZV42000	Peritoneal dialysis bag procedure
Readcode	66i..00	[V]Renal dialysis status
Readcode	6AA..00	[V]Aftercare involving intermittent dialysis
Readcode	9Ot..00	[V]Aftercare involving renal dialysis NOS
Readcode	9Ot0.00	[V]Other specified aftercare involving intermittent dialysis
Readcode	9Ot1.00	[V]Aftercare involving peritoneal dialysis
Readcode	9Ot2.00	[V]Unspecified aftercare involving intermittent dialysis
Readcode	9Ot3.00	[X]Other dialysis
Readcode	9Ot4.00	Transplantation of kidney
ICD-10	N181	Autotransplant of kidney
ICD-10	N182	Transplantation of kidney from live donor
ICD-10	N00	Allotransplantation of kidney from live donor
ICD-10	N10	Transplantation of kidney from cadaver
ICD-10	N17	Allotransplantation of kidney from cadaver
ICD-10	N01	Allotransplantation of kidney from cadaver, heart-beating
ICD-10	N03	Allotransplantation kidney from cadaver, heart non-beating
ICD-10	N052	Other specified transplantation of kidney
ICD-10	N053	Transplantation of kidney NOS
ICD-10	N054	Transplant nephrectomy
ICD-10	N055	Excision of rejected transplanted kidney
ICD-10	N056	Exploration of renal transplant
ICD-10	N072	Interventions associated with transplantation of kidney
ICD-10	N073	Renal tubulo-interstitial disordrs in transplant rejectn
ICD-10	N074	Kidney transplant failure and rejection
ICD-10	N183	Kidney transplant with complication, without blame
ICD-10	N184	Renal transplant with complication, without blame
ICD-10	N185	[V]Kidney transplanted
ICD-10	N189	Chronic kidney disease monitoring
ICD-10	N19	Chronic kidney disease annual review
ICD-10	N25	Chronic kidney disease monitoring administration
ICD-10	Y841	Chronic kidney disease monitoring first letter
ICD-10	Z49	Chronic kidney disease monitoring second letter
ICD-10	Z992	Chronic kidney disease monitoring third letter
ICD-10	T861	Chronic kidney disease monitoring verbal invite
ICD-10	Z940	Chronic kidney disease monitoring telephone invite
Diabetes Mellitus		
Readcode	1434	H/O: diabetes mellitus
Readcode	14F4.00	H/O: Admission in last year for diabetes foot problem
Readcode	14P3.00	H/O: insulin therapy
Readcode	2126300	Diabetes resolved
Readcode	212H.00	Diabetes resolved
Readcode	9OL9.00	Diabetes monitoring deleted
Readcode	13Y1.00	Diabetic association member
Readcode	3881	Education score - diabetes
Readcode	3882	Diabetes well being questionnaire
Readcode	66A..00	Diabetic monitoring
Readcode	66A1.00	Initial diabetic assessment

Readcode	66A2.00	Follow-up diabetic assessment
Readcode	66AM.00	Diabetic - follow-up default
Readcode	66AZ.00	Diabetic monitoring NOS
Readcode	66Af.00	Patient diabetes education review
Readcode	66Ak.00	Diabetic monitoring - lower risk albumin excretion
Readcode	66Al.00	Diabetic monitoring - higher risk albumin excretion
Readcode	679L.00	Health education - diabetes
Readcode	679R.00	Patient offered diabetes structured education programme
Readcode	8A12.00	Diabetic crisis monitoring
Readcode	8A17.00	Self monitoring of blood glucose
Readcode	8A18.00	Self monitoring of urine glucose
Readcode	8A19.00	Self monitoring of blood and urine glucose
Readcode	8A1A.00	Self monitoring urine ketones
Readcode	8CR2.00	Diabetes clinical management plan
Readcode	8CS0.00	Diabetes care plan agreed
Readcode	8HHy.00	Referral to diabetic register
Readcode	8HTe.00	Referral to diabetes preconception counselling clinic
Readcode	8HTk.00	Referral to diabetic eye clinic
Readcode	8Hg4.00	Discharged from care of diabetes specialist nurse
Readcode	8Hj0.00	Referral to diabetes structured education programme
Readcode	8Hj3.00	Referral to DAFNE diabetes structured education programme
Readcode	8Hj4.00	Referral to DESMOND diabetes structured education programme
Readcode	8Hj5.00	Referral to XPERT diabetes structured education programme
Readcode	8I6F.00	Diabetic retinopathy screening not indicated
Readcode	8I6G.00	Diabetic foot examination not indicated
Readcode	8I81.00	Did not complete diabetes structured education programme
Readcode	8I82.00	Did not complete DAFNE diabetes structured education program
Readcode	8I83.00	Did not complete DESMOND diabetes structured educat program
Readcode	8I84.00	Did not complete XPERT diabetes structured education program
Readcode	93C4.00	Patient consent given for addition to diabetic register
Readcode	9N0m.00	Seen in diabetic nurse consultant clinic
Readcode	9N0n.00	Seen in community diabetes specialist clinic
Readcode	9N0o.00	Seen in community diabetic specialist nurse clinic
Readcode	9N1Q.00	Seen in diabetic clinic
Readcode	9N1i.00	Seen in diabetic foot clinic
Readcode	9N1o.00	Seen in multidisciplinary diabetic clinic
Readcode	9N1v.00	Seen in diabetic eye clinic
Readcode	9N2d.00	Seen by diabetologist
Readcode	9N2i.00	Seen by diabetic liaison nurse
Readcode	9N4I.00	DNA - Did not attend diabetic clinic
Readcode	9N4p.00	Did not attend diabetic retinopathy clinic
Readcode	9NM0.00	Attending diabetes clinic
Readcode	9NN8.00	Under care of diabetologist
Readcode	9NN9.00	Under care of diabetes specialist nurse
Readcode	9NND.00	Under care of diabetic foot screener
Readcode	9NiA.00	Did not attend diabetes structured education programme
Readcode	9NiD.00	Did not attend DESMOND diabetes structured education program
Readcode	9NiE.00	Did not attend XPERT diabetes structured education programme
Readcode	9Nl4.00	Seen by general practitioner special interest in diabetes
Readcode	9OL..00	Diabetes monitoring admin.
Readcode	9OL1.00	Attends diabetes monitoring
Readcode	9OL2.00	Refuses diabetes monitoring
Readcode	9OL3.00	Diabetes monitoring default
Readcode	9OL4.00	Diabetes monitoring 1st letter
Readcode	9OL5.00	Diabetes monitoring 2nd letter
Readcode	9OL6.00	Diabetes monitoring 3rd letter
Readcode	9OL7.00	Diabetes monitor.verbal invite
Readcode	9OL8.00	Diabetes monitor.phone invite

Wu J, *et al. Open Heart* 2023; 10:e002357. doi: 10.1136/openhrt-2023-002357

Readcode	9OLA.00	Diabetes monitor. check done
Readcode	9OLA.11	Diabetes monitored
Readcode	9OLB.00	Attended diabetes structured education programme
Readcode	9OLF.00	Diabetes structured education programme completed
Readcode	9OLG.00	Attended XPERT diabetes structured education programme
Readcode	9OLH.00	Attended DAFNE diabetes structured education programme
Readcode	9OLJ.00	DAFNE diabetes structured education programme completed
Readcode	9OLK.00	DESMOND diabetes structured education programme completed
Readcode	9OLL.00	XPERT diabetes structured education programme completed
Readcode	9OLM.00	Diabetes structured education programme declined
Readcode	9OLZ.00	Diabetes monitoring admin.NOS
Readcode	9h4..00	Exception reporting: diabetes quality indicators
Readcode	9h41.00	Excepted from diabetes qual indicators: Patient unsuitable
Readcode	9h42.00	Excepted from diabetes quality indicators: Informed dissent
Readcode	ZL22500	Under care of diabetic liaison nurse
Readcode	ZLA2500	Seen by diabetic liaison nurse
Readcode	ZLD7500	Discharge by diabetic liaison nurse
Readcode	ZRB4.00	Diabetes clinic satisfaction questionnaire
Readcode	ZRB4.11	CSQ - Diabetes clinic satisfaction questionnaire
Readcode	ZRB5.00	Diabetes treatment satisfaction questionnaire
Readcode	ZRB5.11	DTSQ - Diabetes treatment satisfaction questionnaire
Readcode	ZRB6.00	Diabetes wellbeing questionnaire
Readcode	ZRB6.11	DWBQ - Diabetes wellbeing questionnaire
Readcode	ZRBa.00	Education score - diabetes
Readcode	66An.00	Diabetes type 1 review
Readcode	C100000	Diabetes mellitus, juvenile type, no mention of complication
Readcode	C100011	Insulin dependent diabetes mellitus
Readcode	C101000	Diabetes mellitus, juvenile type, with ketoacidosis
Readcode	C102000	Diabetes mellitus, juvenile type, with hyperosmolar coma
Readcode	C103000	Diabetes mellitus, juvenile type, with ketoacidotic coma
Readcode	C104000	Diabetes mellitus, juvenile type, with renal manifestation
Readcode	C105000	Diabetes mellitus, juvenile type, + ophthalmic manifestation
Readcode	C106000	Diabetes mellitus, juvenile, + neurological manifestation
Readcode	C107000	Diabetes mellitus, juvenile +peripheral circulatory disorder
Readcode	C107300	IDDM with peripheral circulatory disorder
Readcode	C108.00	Insulin dependent diabetes mellitus
Readcode	C108.11	IDDM-Insulin dependent diabetes mellitus
Readcode	C108.12	Type 1 diabetes mellitus
Readcode	C108.13	Type I diabetes mellitus
Readcode	C108000	Insulin-dependent diabetes mellitus with renal complications
Readcode	C108011	Type I diabetes mellitus with renal complications
Readcode	C108012	Type 1 diabetes mellitus with renal complications
Readcode	C108100	Insulin-dependent diabetes mellitus with ophthalmic comps
Readcode	C108200	Insulin-dependent diabetes mellitus with neurological comps
Readcode	C108211	Type I diabetes mellitus with neurological complications
Readcode	C108212	Type 1 diabetes mellitus with neurological complications
Readcode	C108300	Insulin dependent diabetes mellitus with multiple complicatn
Readcode	C108400	Unstable insulin dependant diabetes mellitus
Readcode	C108411	Unstable type I diabetes mellitus
Readcode	C108412	Unstable type 1 diabetes mellitus
Readcode	C108500	Insulin dependent diabetes mellitus with ulcer
Readcode	C108511	Type I diabetes mellitus with ulcer
Readcode	C108512	Type 1 diabetes mellitus with ulcer
Readcode	C108600	Insulin dependent diabetes mellitus with gangrene
Readcode	C108700	Insulin dependent diabetes mellitus with retinopathy
Readcode	C108711	Type I diabetes mellitus with retinopathy
Readcode	C108712	Type 1 diabetes mellitus with retinopathy
Readcode	C108800	Insulin dependant diabetes mellitus - poor control

Wu J, *et al. Open Heart* 2023; 10:e002357. doi: 10.1136/openhrt-2023-002357

Readcode	C108811	Type I diabetes mellitus - poor control
Readcode	C108812	Type 1 diabetes mellitus - poor control
Readcode	C108900	Insulin dependant diabetes maturity onset
Readcode	C108911	Type I diabetes mellitus maturity onset
Readcode	C108912	Type 1 diabetes mellitus maturity onset
Readcode	C108A00	Insulin-dependent diabetes without complication
Readcode	C108A11	Type I diabetes mellitus without complication
Readcode	C108B00	Insulin dependent diabetes mellitus with mononeuropathy
Readcode	C108C00	Insulin dependent diabetes mellitus with polyneuropathy
Readcode	C108D00	Insulin dependent diabetes mellitus with nephropathy
Readcode	C108D11	Type I diabetes mellitus with nephropathy
Readcode	C108E00	Insulin dependent diabetes mellitus with hypoglycaemic coma
Readcode	C108E11	Type I diabetes mellitus with hypoglycaemic coma
Readcode	C108E12	Type 1 diabetes mellitus with hypoglycaemic coma
Readcode	C108F00	Insulin dependent diabetes mellitus with diabetic cataract
Readcode	C108F11	Type I diabetes mellitus with diabetic cataract
Readcode	C108G00	Insulin dependent diab mell with peripheral angiopathy
Readcode	C108H00	Insulin dependent diabetes mellitus with arthropathy
Readcode	C108H11	Type I diabetes mellitus with arthropathy
Readcode	C108J00	Insulin dependent diab mell with neuropathic arthropathy
Readcode	C108J11	Type I diabetes mellitus with neuropathic arthropathy
Readcode	C108J12	Type 1 diabetes mellitus with neuropathic arthropathy
Readcode	C10C.12	Maturity onset diabetes in youth type 1
Readcode	C10E.00	Type 1 diabetes mellitus
Readcode	C10E.11	Type I diabetes mellitus
Readcode	C10E.12	Insulin dependent diabetes mellitus
Readcode	C10E000	Type 1 diabetes mellitus with renal complications
Readcode	C10E100	Type 1 diabetes mellitus with ophthalmic complications
Readcode	C10E112	Insulin-dependent diabetes mellitus with ophthalmic comps
Readcode	C10E200	Type 1 diabetes mellitus with neurological complications
Readcode	C10E300	Type 1 diabetes mellitus with multiple complications
Readcode	C10E311	Type I diabetes mellitus with multiple complications
Readcode	C10E312	Insulin dependent diabetes mellitus with multiple complicat
Readcode	C10E400	Unstable type 1 diabetes mellitus
Readcode	C10E411	Unstable type I diabetes mellitus
Readcode	C10E412	Unstable insulin dependent diabetes mellitus
Readcode	C10E500	Type 1 diabetes mellitus with ulcer
Readcode	C10E511	Type I diabetes mellitus with ulcer
Readcode	C10E512	Insulin dependent diabetes mellitus with ulcer
Readcode	C10E600	Type 1 diabetes mellitus with gangrene
Readcode	C10E700	Type 1 diabetes mellitus with retinopathy
Readcode	C10E711	Type I diabetes mellitus with retinopathy
Readcode	C10E712	Insulin dependent diabetes mellitus with retinopathy
Readcode	C10E800	Type 1 diabetes mellitus - poor control
Readcode	C10E812	Insulin dependent diabetes mellitus - poor control
Readcode	C10E900	Type 1 diabetes mellitus maturity onset
Readcode	C10E911	Type I diabetes mellitus maturity onset
Readcode	C10E912	Insulin dependent diabetes maturity onset
Readcode	C10EA00	Type 1 diabetes mellitus without complication
Readcode	C10EA11	Type I diabetes mellitus without complication
Readcode	C10EB00	Type 1 diabetes mellitus with mononeuropathy
Readcode	C10EC00	Type 1 diabetes mellitus with polyneuropathy
Readcode	C10EC11	Type I diabetes mellitus with polyneuropathy
Readcode	C10ED00	Type 1 diabetes mellitus with nephropathy
Readcode	C10EE00	Type 1 diabetes mellitus with hypoglycaemic coma
Readcode	C10EF00	Type 1 diabetes mellitus with diabetic cataract
Readcode	C10EG00	Type 1 diabetes mellitus with peripheral angiopathy
Readcode	C10EH00	Type 1 diabetes mellitus with arthropathy

Readcode	C10EJ00	Type 1 diabetes mellitus with neuropathic arthropathy
Readcode	C10EK00	Type 1 diabetes mellitus with persistent proteinuria
Readcode	C10EL00	Type 1 diabetes mellitus with persistent microalbuminuria
Readcode	C10EM00	Type 1 diabetes mellitus with ketoacidosis
Readcode	C10EM11	Type I diabetes mellitus with ketoacidosis
Readcode	C10EN00	Type 1 diabetes mellitus with ketoacidotic coma
Readcode	C10EN11	Type I diabetes mellitus with ketoacidotic coma
Readcode	C10EP00	Type 1 diabetes mellitus with exudative maculopathy
Readcode	C10EP11	Type I diabetes mellitus with exudative maculopathy
Readcode	C10EQ00	Type 1 diabetes mellitus with gastroparesis
Readcode	C10z000	Diabetes mellitus, juvenile type, + unspecified complication
Readcode	L180500	Pre-existing diabetes mellitus, insulin-dependent
Readcode	M21yC00	Insulin lipohypertrophy
Readcode	M21yC11	Insulin site lipohypertrophy
Readcode	ZC2C900	Dietary advice for type I diabetes
Readcode	ZRbH.00	Perceived control of insulin-dependent diabetes
Readcode	66Ao.00	Diabetes type 2 review
Readcode	C100100	Diabetes mellitus, adult onset, no mention of complication
Readcode	C100111	Maturity onset diabetes
Readcode	C100112	Non-insulin dependent diabetes mellitus
Readcode	C101100	Diabetes mellitus, adult onset, with ketoacidosis
Readcode	C102100	Diabetes mellitus, adult onset, with hyperosmolar coma
Readcode	C103100	Diabetes mellitus, adult onset, with ketoacidotic coma
Readcode	C104100	Diabetes mellitus, adult onset, with renal manifestation
Readcode	C105100	Diabetes mellitus, adult onset, + ophthalmic manifestation
Readcode	C106100	Diabetes mellitus, adult onset, + neurological manifestation
Readcode	C107100	Diabetes mellitus, adult, + peripheral circulatory disorder
Readcode	C107200	Diabetes mellitus, adult with gangrene
Readcode	C107400	NIDDM with peripheral circulatory disorder
Readcode	C109.00	Non-insulin dependent diabetes mellitus
Readcode	C109.11	NIDDM - Non-insulin dependent diabetes mellitus
Readcode	C109.12	Type 2 diabetes mellitus
Readcode	C109.13	Type II diabetes mellitus
Readcode	C109000	Non-insulin-dependent diabetes mellitus with renal comps
Readcode	C109011	Type II diabetes mellitus with renal complications
Readcode	C109012	Type 2 diabetes mellitus with renal complications
Readcode	C109100	Non-insulin-dependent diabetes mellitus with ophthalm comps
Readcode	C109111	Type II diabetes mellitus with ophthalmic complications
Readcode	C109112	Type 2 diabetes mellitus with ophthalmic complications
Readcode	C109200	Non-insulin-dependent diabetes mellitus with neuro comps
Readcode	C109211	Type II diabetes mellitus with neurological complications
Readcode	C109212	Type 2 diabetes mellitus with neurological complications
Readcode	C109300	Non-insulin-dependent diabetes mellitus with multiple comps
Readcode	C109400	Non-insulin dependent diabetes mellitus with ulcer
Readcode	C109411	Type II diabetes mellitus with ulcer
Readcode	C109412	Type 2 diabetes mellitus with ulcer
Readcode	C109500	Non-insulin dependent diabetes mellitus with gangrene
Readcode	C109511	Type II diabetes mellitus with gangrene
Readcode	C109512	Type 2 diabetes mellitus with gangrene
Readcode	C109600	Non-insulin-dependent diabetes mellitus with retinopathy
Readcode	C109611	Type II diabetes mellitus with retinopathy
Readcode	C109612	Type 2 diabetes mellitus with retinopathy
Readcode	C109700	Non-insulin dependant diabetes mellitus - poor control
Readcode	C109711	Type II diabetes mellitus - poor control
Readcode	C109712	Type 2 diabetes mellitus - poor control
Readcode	C109900	Non-insulin-dependent diabetes mellitus without complication
Readcode	C109A00	Non-insulin dependent diabetes mellitus with mononeuropathy
Readcode	C109A11	Type II diabetes mellitus with mononeuropathy

Readcode	C109B00	Non-insulin dependent diabetes mellitus with polyneuropathy
Readcode	C109B11	Type II diabetes mellitus with polyneuropathy
Readcode	C109C00	Non-insulin dependent diabetes mellitus with nephropathy
Readcode	C109C11	Type II diabetes mellitus with nephropathy
Readcode	C109C12	Type 2 diabetes mellitus with nephropathy
Readcode	C109D00	Non-insulin dependent diabetes mellitus with hypoglyca coma
Readcode	C109D11	Type II diabetes mellitus with hypoglycaemic coma
Readcode	C109D12	Type 2 diabetes mellitus with hypoglycaemic coma
Readcode	C109E00	Non-insulin depend diabetes mellitus with diabetic cataract
Readcode	C109E11	Type II diabetes mellitus with diabetic cataract
Readcode	C109E12	Type 2 diabetes mellitus with diabetic cataract
Readcode	C109F00	Non-insulin-dependent d m with peripheral angiopath
Readcode	C109F11	Type II diabetes mellitus with peripheral angiopathy
Readcode	C109F12	Type 2 diabetes mellitus with peripheral angiopathy
Readcode	C109G00	Non-insulin dependent diabetes mellitus with arthropathy
Readcode	C109G11	Type II diabetes mellitus with arthropathy
Readcode	C109G12	Type 2 diabetes mellitus with arthropathy
Readcode	C109H00	Non-insulin dependent d m with neuropathic arthropathy
Readcode	C109H11	Type II diabetes mellitus with neuropathic arthropathy
Readcode	C109H12	Type 2 diabetes mellitus with neuropathic arthropathy
Readcode	C109J00	Insulin treated Type 2 diabetes mellitus
Readcode	C109J11	Insulin treated non-insulin dependent diabetes mellitus
Readcode	C109J12	Insulin treated Type II diabetes mellitus
Readcode	C109K00	Hyperosmolar non-ketotic state in type 2 diabetes mellitus
Readcode	C10C.11	Maturity onset diabetes in youth
Readcode	C10D.00	Diabetes mellitus autosomal dominant type 2
Readcode	C10D.11	Maturity onset diabetes in youth type 2
Readcode	C10ER00	Latent autoimmune diabetes mellitus in adult
Readcode	C10F.00	Type 2 diabetes mellitus
Readcode	C10F.11	Type II diabetes mellitus
Readcode	C10F000	Type 2 diabetes mellitus with renal complications
Readcode	C10F011	Type II diabetes mellitus with renal complications
Readcode	C10F100	Type 2 diabetes mellitus with ophthalmic complications
Readcode	C10F200	Type 2 diabetes mellitus with neurological complications
Readcode	C10F211	Type II diabetes mellitus with neurological complications
Readcode	C10F300	Type 2 diabetes mellitus with multiple complications
Readcode	C10F311	Type II diabetes mellitus with multiple complications
Readcode	C10F400	Type 2 diabetes mellitus with ulcer
Readcode	C10F411	Type II diabetes mellitus with ulcer
Readcode	C10F500	Type 2 diabetes mellitus with gangrene
Readcode	C10F600	Type 2 diabetes mellitus with retinopathy
Readcode	C10F611	Type II diabetes mellitus with retinopathy
Readcode	C10F700	Type 2 diabetes mellitus - poor control
Readcode	C10F711	Type II diabetes mellitus - poor control
Readcode	C10F900	Type 2 diabetes mellitus without complication
Readcode	C10F911	Type II diabetes mellitus without complication
Readcode	C10FA00	Type 2 diabetes mellitus with mononeuropathy
Readcode	C10FA11	Type II diabetes mellitus with mononeuropathy
Readcode	C10FB00	Type 2 diabetes mellitus with polyneuropathy
Readcode	C10FB11	Type II diabetes mellitus with polyneuropathy
Readcode	C10FC00	Type 2 diabetes mellitus with nephropathy
Readcode	C10FD00	Type 2 diabetes mellitus with hypoglycaemic coma
Readcode	C10FD11	Type II diabetes mellitus with hypoglycaemic coma
Readcode	C10FE00	Type 2 diabetes mellitus with diabetic cataract
Readcode	C10FE11	Type II diabetes mellitus with diabetic cataract
Readcode	C10FF00	Type 2 diabetes mellitus with peripheral angiopathy
Readcode	C10FG00	Type 2 diabetes mellitus with arthropathy
Readcode	C10FH00	Type 2 diabetes mellitus with neuropathic arthropathy

Readcode	C10FJ00	Insulin treated Type 2 diabetes mellitus
Readcode	C10FJ11	Insulin treated Type II diabetes mellitus
Readcode	C10FK00	Hyperosmolar non-ketotic state in type 2 diabetes mellitus
Readcode	C10FL00	Type 2 diabetes mellitus with persistent proteinuria
Readcode	C10FL11	Type II diabetes mellitus with persistent proteinuria
Readcode	C10FM00	Type 2 diabetes mellitus with persistent microalbuminuria
Readcode	C10FM11	Type II diabetes mellitus with persistent microalbuminuria
Readcode	C10FN00	Type 2 diabetes mellitus with ketoacidosis
Readcode	C10FP00	Type 2 diabetes mellitus with ketoacidotic coma
Readcode	C10FQ00	Type 2 diabetes mellitus with exudative maculopathy
Readcode	C10FR00	Type 2 diabetes mellitus with gastroparesis
Readcode	C10y100	Diabetes mellitus, adult, + other specified manifestation
Readcode	C10z100	Diabetes mellitus, adult onset, + unspecified complication
Readcode	L180600	Pre-existing diabetes mellitus, non-insulin-dependent
Readcode	ZC2CA00	Dietary advice for type II diabetes
Readcode	C10B.00	Diabetes mellitus induced by steroids
Readcode	C10B000	Steroid induced diabetes mellitus without complication
Readcode	C10FS00	Maternally inherited diabetes mellitus
Readcode	C10G.00	Secondary pancreatic diabetes mellitus
Readcode	C10G000	Secondary pancreatic diabetes mellitus without complication
Readcode	C10H.00	Diabetes mellitus induced by non-steroid drugs
Readcode	C10H000	DM induced by non-steroid drugs without complication
Readcode	C10N.00	Secondary diabetes mellitus
Readcode	C10N000	Secondary diabetes mellitus without complication
Readcode	C10N100	Cystic fibrosis related diabetes mellitus
Readcode	C11y000	Steroid induced diabetes
Readcode	13AB.00	Diabetic lipid lowering diet
Readcode	13AC.00	Diabetic weight reducing diet
Readcode	13B1.00	Diabetic diet
Readcode	2BBF.00	Retinal abnormality - diabetes related
Readcode	2BBL.00	O/E - diabetic maculopathy present both eyes
Readcode	2BBM.00	O/E - diabetic maculopathy absent both eyes
Readcode	2BBP.00	O/E - right eye background diabetic retinopathy
Readcode	2BBQ.00	O/E - left eye background diabetic retinopathy
Readcode	2BBR.00	O/E - right eye preproliferative diabetic retinopathy
Readcode	2BBS.00	O/E - left eye preproliferative diabetic retinopathy
Readcode	2BBT.00	O/E - right eye proliferative diabetic retinopathy
Readcode	2BBV.00	O/E - left eye proliferative diabetic retinopathy
Readcode	2BBW.00	O/E - right eye diabetic maculopathy
Readcode	2BBX.00	O/E - left eye diabetic maculopathy
Readcode	2BBk.00	O/E - right eye stable treated prolif diabetic retinopathy
Readcode	2BBl.00	O/E - left eye stable treated prolif diabetic retinopathy
Readcode	2BBo.00	O/E - sight threatening diabetic retinopathy
Readcode	2G51000	Foot abnormality - diabetes related
Readcode	2G5A.00	O/E - Right diabetic foot at risk
Readcode	2G5B.00	O/E - Left diabetic foot at risk
Readcode	2G5C.00	Foot abnormality - diabetes related
Readcode	2G5E.00	O/E - Right diabetic foot at low risk
Readcode	2G5F.00	O/E - Right diabetic foot at moderate risk
Readcode	2G5G.00	O/E - Right diabetic foot at high risk
Readcode	2G5H.00	O/E - Right diabetic foot - ulcerated
Readcode	2G5I.00	O/E - Left diabetic foot at low risk
Readcode	2G5J.00	O/E - Left diabetic foot at moderate risk
Readcode	2G5K.00	O/E - Left diabetic foot at high risk
Readcode	2G5L.00	O/E - Left diabetic foot - ulcerated
Readcode	2G5V.00	O/E - right chronic diabetic foot ulcer
Readcode	2G5W.00	O/E - left chronic diabetic foot ulcer
Readcode	66A3.00	Diabetic on diet only

Wu J, *et al. Open Heart* 2023; 10:e002357. doi: 10.1136/openhrt-2023-002357

Readcode	66A4.00	Diabetic on oral treatment
Readcode	66A5.00	Diabetic on insulin
Readcode	66A8.00	Has seen dietician - diabetes
Readcode	66A9.00	Understands diet - diabetes
Readcode	66AA.11	Injection sites - diabetic
Readcode	66AD.00	Fundoscopy - diabetic check
Readcode	66AG.00	Diabetic drug side effects
Readcode	66AH.00	Diabetic treatment changed
Readcode	66AI.00	Diabetic - good control
Readcode	66AJ.00	Diabetic - poor control
Readcode	66AJ.11	Unstable diabetes
Readcode	66AJ100	Brittle diabetes
Readcode	66AJz00	Diabetic - poor control NOS
Readcode	66AK.00	Diabetic - cooperative patient
Readcode	66AL.00	Diabetic-uncooperative patient
Readcode	66AN.00	Date diabetic treatment start
Readcode	66AO.00	Date diabetic treatment stopp.
Readcode	66AP.00	Diabetes: practice programme
Readcode	66AQ.00	Diabetes: shared care programme
Readcode	66AR.00	Diabetes management plan given
Readcode	66AS.00	Diabetic annual review
Readcode	66AT.00	Annual diabetic blood test
Readcode	66AU.00	Diabetes care by hospital only
Readcode	66AV.00	Diabetic on insulin and oral treatment
Readcode	66AW.00	Diabetic foot risk assessment
Readcode	66AX.00	Diabetes: shared care in pregnancy - diabetol and obstet
Readcode	66AY.00	Diabetic diet - good compliance
Readcode	66Aa.00	Diabetic diet - poor compliance
Readcode	66Ab.00	Diabetic foot examination
Readcode	66Ac.00	Diabetic peripheral neuropathy screening
Readcode	66Ag.00	Insulin needles changed daily
Readcode	66Ah.00	Insulin needles changed for each injection
Readcode	66Ai.00	Diabetic 6 month review
Readcode	66Aj.00	Insulin needles changed less than once a day
Readcode	66Am.00	Insulin dose changed
Readcode	66Ap.00	Insulin treatment initiated
Readcode	66Aq.00	Diabetic foot screen
Readcode	6761	Diabetic pre-pregnancy counselling
Readcode	68A7.00	Diabetic retinopathy screening
Readcode	68A9.00	Diabetic retinopathy screening offered
Readcode	68AB.00	Diabetic digital retinopathy screening offered
Readcode	7276	Pan retinal photocoagulation for diabetes
Readcode	7L10000	Continuous subcutaneous infusion of insulin
Readcode	7L19800	Subcutaneous injection of insulin
Readcode	889A.00	Diab mellit insulin-glucose infus acute myocardial infarct
Readcode	8A13.00	Diabetic stabilisation
Readcode	8B3l.00	Diabetes medication review
Readcode	8BL2.00	Patient on maximal tolerated therapy for diabetes
Readcode	8CA4100	Pt advised re diabetic diet
Readcode	8CAQ.00	Advice about blood glucose control
Readcode	8CP2.00	Transition of diabetes care options discussed
Readcode	8H2J.00	Admit diabetic emergency
Readcode	8H3O.00	Non-urgent diabetic admission
Readcode	8H7r.00	Refer to diabetic foot screener
Readcode	8HBG.00	Diabetic retinopathy 12 month review
Readcode	8HBH.00	Diabetic retinopathy 6 month review
Readcode	8HLE.00	Diabetology D.V. done
Readcode	8Hl1.00	Referral for diabetic retinopathy screening

Readcode	8I3W.00	Diabetic foot examination declined
Readcode	8I3X.00	Diabetic retinopathy screening refused
Readcode	8I3k.00	Insulin therapy declined
Readcode	8I57.00	Patient held diabetic record declined
Readcode	9360	Patient held diabetic record issued
Readcode	9OLD.00	Diabetic patient unsuitable for digital retinal photography
Readcode	C10..00	Diabetes mellitus
Readcode	C100.00	Diabetes mellitus with no mention of complication
Readcode	C100z00	Diabetes mellitus NOS with no mention of complication
Readcode	C101.00	Diabetes mellitus with ketoacidosis
Readcode	C101y00	Other specified diabetes mellitus with ketoacidosis
Readcode	C101z00	Diabetes mellitus NOS with ketoacidosis
Readcode	C102.00	Diabetes mellitus with hyperosmolar coma
Readcode	C102z00	Diabetes mellitus NOS with hyperosmolar coma
Readcode	C103.00	Diabetes mellitus with ketoacidotic coma
Readcode	C103y00	Other specified diabetes mellitus with coma
Readcode	C103z00	Diabetes mellitus NOS with ketoacidotic coma
Readcode	C104.00	Diabetes mellitus with renal manifestation
Readcode	C104.11	Diabetic nephropathy
Readcode	C104y00	Other specified diabetes mellitus with renal complications
Readcode	C104z00	Diabetes mellitis with nephropathy NOS
Readcode	C105.00	Diabetes mellitus with ophthalmic manifestation
Readcode	C105y00	Other specified diabetes mellitus with ophthalmic complicatn
Readcode	C105z00	Diabetes mellitus NOS with ophthalmic manifestation
Readcode	C106.00	Diabetes mellitus with neurological manifestation
Readcode	C106.11	Diabetic amyotrophy
Readcode	C106.12	Diabetes mellitus with neuropathy
Readcode	C106.13	Diabetes mellitus with polyneuropathy
Readcode	C106y00	Other specified diabetes mellitus with neurological comps
Readcode	C106z00	Diabetes mellitus NOS with neurological manifestation
Readcode	C107.00	Diabetes mellitus with peripheral circulatory disorder
Readcode	C107.11	Diabetes mellitus with gangrene
Readcode	C107.12	Diabetes with gangrene
Readcode	C107z00	Diabetes mellitus NOS with peripheral circulatory disorder
Readcode	C108y00	Other specified diabetes mellitus with multiple comps
Readcode	C108z00	Unspecified diabetes mellitus with multiple complications
Readcode	C10A.00	Malnutrition-related diabetes mellitus
Readcode	C10A000	Malnutrition-related diabetes mellitus with coma
Readcode	C10A100	Malnutrition-related diabetes mellitus with ketoacidosis
Readcode	C10C.00	Diabetes mellitus autosomal dominant
Readcode	C10M.00	Lipoatrophic diabetes mellitus
Readcode	C10y.00	Diabetes mellitus with other specified manifestation
Readcode	C10yy00	Other specified diabetes mellitus with other spec comps
Readcode	C10yz00	Diabetes mellitus NOS with other specified manifestation
Readcode	C10z.00	Diabetes mellitus with unspecified complication
Readcode	C10zy00	Other specified diabetes mellitus with unspecified comps
Readcode	C10zz00	Diabetes mellitus NOS with unspecified complication
Readcode	C314.11	Renal diabetes
Readcode	C350011	Bronzed diabetes
Readcode	Cyu2.00	[X]Diabetes mellitus
Readcode	Cyu2000	[X]Other specified diabetes mellitus
Readcode	F171100	Autonomic neuropathy due to diabetes
Readcode	F345000	Diabetic mononeuritis multiplex
Readcode	F35z000	Diabetic mononeuritis NOS
Readcode	F372.00	Polyneuropathy in diabetes
Readcode	F372.11	Diabetic polyneuropathy
Readcode	F372.12	Diabetic neuropathy
Readcode	F372000	Acute painful diabetic neuropathy

Readcode	F372100	Chronic painful diabetic neuropathy
Readcode	F372200	Asymptomatic diabetic neuropathy
Readcode	F381300	Myasthenic syndrome due to diabetic amyotrophy
Readcode	F381311	Diabetic amyotrophy
Readcode	F3y0.00	Diabetic mononeuropathy
Readcode	F420.00	Diabetic retinopathy
Readcode	F420000	Background diabetic retinopathy
Readcode	F420100	Proliferative diabetic retinopathy
Readcode	F420200	Preproliferative diabetic retinopathy
Readcode	F420300	Advanced diabetic maculopathy
Readcode	F420400	Diabetic maculopathy
Readcode	F420500	Advanced diabetic retinal disease
Readcode	F420600	Non proliferative diabetic retinopathy
Readcode	F420700	High risk proliferative diabetic retinopathy
Readcode	F420800	High risk non proliferative diabetic retinopathy
Readcode	F420z00	Diabetic retinopathy NOS
Readcode	F440700	Diabetic iritis
Readcode	F464000	Diabetic cataract
Readcode	G73y000	Diabetic peripheral angiopathy
Readcode	K01x100	Nephrotic syndrome in diabetes mellitus
Readcode	K01x111	Kimmelstiel - Wilson disease
Readcode	L180X00	Pre-existing diabetes mellitus, unspecified
Readcode	M037200	Cellulitis in diabetic foot
Readcode	M271000	Ischaemic ulcer diabetic foot
Readcode	M271100	Neuropathic diabetic ulcer - foot
Readcode	M271200	Mixed diabetic ulcer - foot
Readcode	N030000	Diabetic cheiroarthropathy
Readcode	N030011	Diabetic cheiropathy
Readcode	N030100	Diabetic Charcot arthropathy
Readcode	Q441.00	Neonatal diabetes mellitus
Readcode	R054200	[D]Gangrene of toe in diabetic
Readcode	R054300	[D]Widespread diabetic foot gangrene
Readcode	TJ23.00	Adverse reaction to insulins and antidiabetic agents
Readcode	TJ23z00	Adverse reaction to insulins and antidiabetic agents NOS
Readcode	U602311	[X] Adverse reaction to insulins and antidiabetic agents
Readcode	ZC2C800	Dietary advice for diabetes mellitus
Readcode	ZV65312	[V]Dietary counselling in diabetes mellitus
Readcode	1I0..00	Diabetes mellitus excluded
ICD-10	E10	Insulin-dependent diabetes mellitus
ICD-10	E11	Non-insulin-dependent diabetes mellitus
ICD-10	E12	Malnutrition-related diabetes mellitus
ICD-10	O242	Diabetes mellitus in pregnancy: Pre-existing malnutrition-related diabetes mellitus
ICD-10	E13	Other specified diabetes mellitus
ICD-10	E14	Unspecified diabetes mellitus
ICD-10	G590	Diabetic mononeuropathy
ICD-10	G632	Diabetic polyneuropathy
ICD-10	H280	Diabetic cataract
ICD-10	H360	Diabetic retinopathy
ICD-10	M142	Diabetic arthropathy
ICD-10	N083	Glomerular disorders in diabetes mellitus
ICD-10	O240	Diabetes mellitus in pregnancy: Pre-existing diabetes mellitus, insulin-dependent
ICD-10	O241	Diabetes mellitus in pregnancy: Pre-existing diabetes mellitus, non-insulin-dependent
ICD-10	O243	Diabetes mellitus in pregnancy: Pre-existing diabetes mellitus, unspecified
Heart Failure		
Readcode	14A6.00	H/O: heart failure
Readcode	14AM.00	H/O: Heart failure in last year
Readcode	1736	Paroxysmal nocturnal dyspnoea

Readcode	1J60.00	Suspected heart failure
Readcode	23E1.00	O/E - pulmonary oedema
Readcode	388D.00	New York Heart Assoc classification heart failure symptoms
Readcode	662T.00	Congestive heart failure monitoring
Readcode	662f.00	New York Heart Association classification - class I
Readcode	662g.00	New York Heart Association classification - class II
Readcode	662h.00	New York Heart Association classification - class III
Readcode	662i.00	New York Heart Association classification - class IV
Readcode	679X.00	Heart failure education
Readcode	8CL3.00	Heart failure care plan discussed with patient
Readcode	8HBE.00	Heart failure follow-up
Readcode	8HHz.00	Referral to heart failure exercise programme
Readcode	8Hg8.00	Discharge from practice nurse heart failure clinic
Readcode	8Hk0.00	Referred to heart failure education group
Readcode	9N0k.00	Seen in heart failure clinic
Readcode	9N2p.00	Seen by community heart failure nurse
Readcode	9N4s.00	Did not attend practice nurse heart failure clinic
Readcode	9N4w.00	Did not attend heart failure clinic
Readcode	9N6T.00	Referred by heart failure nurse specialist
Readcode	9On..00	Left ventricular dysfunction monitoring administration
Readcode	9On0.00	Left ventricular dysfunction monitoring first letter
Readcode	9On1.00	Left ventricular dysfunction monitoring second letter
Readcode	9On2.00	Left ventricular dysfunction monitoring third letter
Readcode	9On3.00	Left ventricular dysfunction monitoring verbal invite
Readcode	9On4.00	Left ventricular dysfunction monitoring telephone invite
Readcode	9Or..00	Heart failure monitoring administration
Readcode	9Or1.00	Heart failure monitoring telephone invite
Readcode	9Or2.00	Heart failure monitoring verbal invite
Readcode	9Or3.00	Heart failure monitoring first letter
Readcode	9Or4.00	Heart failure monitoring second letter
Readcode	9Or5.00	Heart failure monitoring third letter
Readcode	9h1..00	Exception reporting: LVD quality indicators
Readcode	9h11.00	Excepted from LVD quality indicators: Patient unsuitable
Readcode	9h12.00	Excepted from LVD quality indicators: Informed dissent
Readcode	9hH..00	Exception reporting: heart failure quality indicators
Readcode	9hH0.00	Excepted heart failure quality indicators: Patient unsuitabl
Readcode	9hH1.00	Excepted heart failure quality indicators: Informed dissent
Readcode	G581.12	Pulmonary oedema - acute
Readcode	G58z.11	Weak heart
Readcode	H54..00	Pulmonary congestion and hypostasis
Readcode	H541.00	Pulmonary congestion
Readcode	H541000	Chronic pulmonary oedema
Readcode	H541z00	Pulmonary oedema NOS
Readcode	H54z.00	Pulmonary congestion and hypostasis NOS
Readcode	H584.00	Acute pulmonary oedema unspecified
Readcode	H584z00	Acute pulmonary oedema NOS
Readcode	ZRad.00	New York Heart Assoc classification heart failure symptoms
Readcode	G580400	Congestive heart failure due to valvular disease
Readcode	G210.00	Malignant hypertensive heart disease
Readcode	G210000	Malignant hypertensive heart disease without CCF
Readcode	G210100	Malignant hypertensive heart disease with CCF
Readcode	G211100	Benign hypertensive heart disease with CCF
Readcode	G21z100	Hypertensive heart disease NOS with CCF
Readcode	G230.00	Malignant hypertensive heart and renal disease
Readcode	G232.00	Hypertensive heart&renal dis wth (congestive) heart failure
Readcode	G234.00	Hyperten heart&renal dis+both(congestv)heart and renal fail
Readcode	G1yz100	Rheumatic left ventricular failure
Readcode	1O1..00	Heart failure confirmed

Readcode	662W.00	Heart failure annual review
Readcode	662p.00	Heart failure 6 month review
Readcode	8B29.00	Cardiac failure therapy
Readcode	8H2S.00	Admit heart failure emergency
Readcode	9Or0.00	Heart failure review completed
Readcode	G400.00	Acute cor pulmonale
Readcode	G41z.11	Chronic cor pulmonale
Readcode	G554000	Congestive cardiomyopathy
Readcode	G554011	Congestive obstructive cardiomyopathy
Readcode	G58..00	Heart failure
Readcode	G58..11	Cardiac failure
Readcode	G580.00	Congestive heart failure
Readcode	G580.11	Congestive cardiac failure
Readcode	G580.12	Right heart failure
Readcode	G580.13	Right ventricular failure
Readcode	G580.14	Biventricular failure
Readcode	G580000	Acute congestive heart failure
Readcode	G580100	Chronic congestive heart failure
Readcode	G580200	Decompensated cardiac failure
Readcode	G580300	Compensated cardiac failure
Readcode	G581.00	Left ventricular failure
Readcode	G581.11	Asthma - cardiac
Readcode	G581.13	Impaired left ventricular function
Readcode	G581000	Acute left ventricular failure
Readcode	G582.00	Acute heart failure
Readcode	G58z.00	Heart failure NOS
Readcode	G58z.12	Cardiac failure NOS
Readcode	G5yy900	Left ventricular systolic dysfunction
Readcode	G5yyA00	Left ventricular diastolic dysfunction
Readcode	R2y1000	[D]Cardiorespiratory failure
Readcode	Q48y100	Congenital cardiac failure
ICD-10	I110	Hypertensive heart disease with (congestive) heart failure
ICD-10	I130	Hypertensive heart and renal disease with (congestive) heart failure
ICD-10	I132	Hypertensive heart and renal disease with both (congestive) heart failure and renal failure
ICD-10	I260	Pulmonary embolism with mention of acute cor pulmonale
ICD-10	I50	Heart failure
Myocardial Infarction		
Readcode	14A3.00	H/O: myocardial infarct <60
Readcode	14A4.00	H/O: myocardial infarct >60
Readcode	14AH.00	H/O: Myocardial infarction in last year
Readcode	G310.00	Postmyocardial infarction syndrome
Readcode	G32..00	Old myocardial infarction
Readcode	G32..11	Healed myocardial infarction
Readcode	G32..12	Personal history of myocardial infarction
Readcode	G33z500	Post infarct angina
Readcode	G30..11	Attack - heart
Readcode	G30..14	Heart attack
Readcode	G30..17	Silent myocardial infarction
Readcode	G30A.00	Mural thrombosis
Readcode	G30X000	Acute ST segment elevation myocardial infarction
Readcode	G307100	Acute non-ST segment elevation myocardial infarction
Readcode	323..00	ECG: myocardial infarction
Readcode	3233	ECG: antero-septal infarct.
Readcode	3234	ECG:posterior/inferior infarct
Readcode	3235	ECG: subendocardial infarct
Readcode	3236	ECG: lateral infarction
Readcode	323Z.00	ECG: myocardial infarct NOS

Readcode	889A.00	Diab mellit insulin-glucose infus acute myocardial infarct
Readcode	G30..00	Acute myocardial infarction
Readcode	G30..12	Coronary thrombosis
Readcode	G30..13	Cardiac rupture following myocardial infarction (MI)
Readcode	G30..15	MI - acute myocardial infarction
Readcode	G30..16	Thrombosis - coronary
Readcode	G300.00	Acute anterolateral infarction
Readcode	G301.00	Other specified anterior myocardial infarction
Readcode	G301000	Acute anteroapical infarction
Readcode	G301100	Acute anteroseptal infarction
Readcode	G301z00	Anterior myocardial infarction NOS
Readcode	G302.00	Acute inferolateral infarction
Readcode	G303.00	Acute inferoposterior infarction
Readcode	G304.00	Posterior myocardial infarction NOS
Readcode	G305.00	Lateral myocardial infarction NOS
Readcode	G306.00	True posterior myocardial infarction
Readcode	G307.00	Acute subendocardial infarction
Readcode	G307000	Acute non-Q wave infarction
Readcode	G308.00	Inferior myocardial infarction NOS
Readcode	G309.00	Acute Q-wave infarct
Readcode	G30B.00	Acute posterolateral myocardial infarction
Readcode	G30X.00	Acute transmural myocardial infarction of unspecif site
Readcode	G30y.00	Other acute myocardial infarction
Readcode	G30y000	Acute atrial infarction
Readcode	G30y100	Acute papillary muscle infarction
Readcode	G30y200	Acute septal infarction
Readcode	G30yz00	Other acute myocardial infarction NOS
Readcode	G30z.00	Acute myocardial infarction NOS
Readcode	G31y100	Microinfarction of heart
Readcode	G38..00	Postoperative myocardial infarction
Readcode	G380.00	Postoperative transmural myocardial infarction anterior wall
Readcode	G381.00	Postoperative transmural myocardial infarction inferior wall
Readcode	G384.00	Postoperative subendocardial myocardial infarction
Readcode	G38z.00	Postoperative myocardial infarction, unspecified
Readcode	Gyu3400	[X]Acute transmural myocardial infarction of unspecif site
Readcode	G35..00	Subsequent myocardial infarction
Readcode	G350.00	Subsequent myocardial infarction of anterior wall
Readcode	G351.00	Subsequent myocardial infarction of inferior wall
Readcode	G353.00	Subsequent myocardial infarction of other sites
Readcode	G35X.00	Subsequent myocardial infarction of unspecified site
Readcode	G310.11	Dressler's syndrome
Readcode	G36..00	Certain current complication follow acute myocardial infarct
Readcode	G360.00	Haemopericardium/current comp folow acut myocard infarct
Readcode	G361.00	Atrial septal defect/curr comp folow acut myocardal infarct
Readcode	G362.00	Ventric septal defect/curr comp fol acut myocardal infarctn
Readcode	G363.00	Ruptur cardiac wall w'out haemopericard/cur comp fol ac MI
Readcode	G364.00	Ruptur chordae tendinae/curr comp fol acute myocard infarct
Readcode	G365.00	Rupture papillary muscle/curr comp fol acute myocard infarct
Readcode	G366.00	Thrombosis atrium,auric append&vent/curr comp foll acute MI
Readcode	G501.00	Post infarction pericarditis
ICD-10	I252	Old myocardial infarction
ICD-10	I21	Acute myocardial infarction
ICD-10	I22	Subsequent myocardial infarction
ICD-10	I23	Certain current complications following acute myocardial infarction
ICD-10	I241	Dressler's syndrome
Peripheral Vascular Disease		
Readcode	G73..12	Ischaemia of legs
Readcode	G73zz00	Peripheral vascular disease NOS

Readcode	G73z.00	Peripheral vascular disease NOS
Readcode	G670.11	Precerebral atherosclerosis
Readcode	G73..11	Peripheral ischaemic vascular disease
Readcode	G73..00	Other peripheral vascular disease
Readcode	G73..13	Peripheral ischaemia
Readcode	G670.00	Cerebral atherosclerosis
Readcode	24EA.00	O/E - Absent right foot pulses
Readcode	24EC.00	O/E - Right dorsalis pedis abnormal
Readcode	24E9.00	O/E - R.dorsalis pedis absent
Readcode	24F9.00	O/E - L.dorsalis pedis absent
Readcode	M271000	Ischaemic ulcer diabetic foot
Readcode	C107.11	Diabetes mellitus with gangrene
Readcode	C107.12	Diabetes with gangrene
Readcode	C107.00	Diabetes mellitus with peripheral circulatory disorder
Readcode	C109F11	Type II diabetes mellitus with peripheral angiopathy
Readcode	C108600	Insulin dependent diabetes mellitus with gangrene
Readcode	C10E611	Type I diabetes mellitus with gangrene
Readcode	C10FF11	Type II diabetes mellitus with peripheral angiopathy
ICD-10	I731	Thromboangiitis obliterans [Buerger]
ICD-10	I738	Other specified peripheral vascular diseases
ICD-10	I739	Peripheral vascular disease, unspecified
Stroke/Transient Ischaemic Attack		
Readcode	14AB.00	H/O: TIA
Readcode	G65z000	Impending cerebral ischaemia
Readcode	G65z100	Intermittent cerebral ischaemia
Readcode	Fyu5500	[X]Other transnt cerebral ischaemic attacks+related syndroms
Readcode	G65..00	Transient cerebral ischaemia
Readcode	G65..12	Transient ischaemic attack
Readcode	G65y.00	Other transient cerebral ischaemia
Readcode	G65z.00	Transient cerebral ischaemia NOS
Readcode	G65zz00	Transient cerebral ischaemia NOS
Readcode	G683.00	Sequelae of cerebral infarction
Readcode	G64..11	CVA - cerebral artery occlusion
Readcode	G64..13	Stroke due to cerebral arterial occlusion
Readcode	G671.00	Generalised ischaemic cerebrovascular disease NOS
Readcode	G6W..00	Cereb infarct due unsp occlus/stenos precerebr arteries
Readcode	G6X..00	Cerebrl infarctn due/unspcf occlusn or sten/cerebrl artrs
Readcode	Gyu6300	[X]Cerebrl infarctn due/unspcf occlusn or sten/cerebrl artrs
Readcode	Gyu6400	[X]Other cerebral infarction
Readcode	Gyu6500	[X]Occlusion and stenosis of other precerebral arteries
Readcode	Gyu6600	[X]Occlusion and stenosis of other cerebral arteries
Readcode	Gyu6G00	[X]Cereb infarct due unsp occlus/stenos precerebr arteries
Readcode	662o.00	Haemorrhagic stroke monitoring
Readcode	G681.00	Sequelae of intracerebral haemorrhage
Readcode	G682.00	Sequelae of other nontraumatic intracranial haemorrhage
Readcode	G61..00	Intracerebral haemorrhage
Readcode	G61..11	CVA - cerebrovascular accid due to intracerebral haemorrhage
Readcode	G61..12	Stroke due to intracerebral haemorrhage
Readcode	G610.00	Cortical haemorrhage
Readcode	G611.00	Internal capsule haemorrhage
Readcode	G612.00	Basal nucleus haemorrhage
Readcode	G613.00	Cerebellar haemorrhage
Readcode	G614.00	Pontine haemorrhage
Readcode	G616.00	External capsule haemorrhage
Readcode	G617.00	Intracerebral haemorrhage, intraventricular
Readcode	G618.00	Intracerebral haemorrhage, multiple localized
Readcode	G61X.00	Intracerebral haemorrhage in hemisphere, unspecified
Readcode	G61X000	Left sided intracerebral haemorrhage, unspecified

Readcode	G61X100	Right sided intracerebral haemorrhage, unspecified
Readcode	G61z.00	Intracerebral haemorrhage NOS
Readcode	Gyu6200	[X]Other intracerebral haemorrhage
Readcode	Gyu6F00	[X]Intracerebral haemorrhage in hemisphere, unspecified
Readcode	G601.00	Subarachnoid haemorrhage from carotid siphon and bifurcation
Readcode	G602.00	Subarachnoid haemorrhage from middle cerebral artery
Readcode	G60X.00	Subarachnoid haemorrh from intracranial artery, unspecif
Readcode	7017000	Evacuation of subdural haematoma
Readcode	G621.00	Subdural haemorrhage - nontraumatic
Readcode	G622.00	Subdural haematoma - nontraumatic
Readcode	G623.00	Subdural haemorrhage NOS
Readcode	S62..13	Subdural haemorrhage following injury
Readcode	S622.00	Closed traumatic subdural haemorrhage
Readcode	S623.00	Open traumatic subdural haemorrhage
Readcode	S628.00	Traumatic subdural haemorrhage
Readcode	S629.00	Traumatic subdural haematoma
Readcode	S629000	Traumatic subdural haematoma without open intracranial wound
Readcode	S629100	Traumatic subdural haematoma with open intracranial wound
Readcode	7032000	Evacuation of extradural haematoma
Readcode	G620.00	Extradural haemorrhage - nontraumatic
Readcode	S62..11	Extradural haemorrhage following injury
Readcode	S624.00	Closed traumatic extradural haemorrhage
Readcode	S624.11	Epidural haematoma following injury
Readcode	S625.00	Open traumatic extradural haemorrhage
Readcode	S626.00	Epidural haemorrhage
Readcode	S62A.00	Traumatic extradural haematoma
Readcode	G62..00	Other and unspecified intracranial haemorrhage
Readcode	G62z.00	Intracranial haemorrhage NOS
Readcode	A94y600	Rupture of syphilitic cerebral aneurysm
Readcode	S62..00	Cerebral haemorrhage following injury
Readcode	S62..14	Traumatic cerebral haemorrhage
Readcode	S62z.00	Cerebral haemorrhage following injury NOS
Readcode	S63..00	Other cerebral haemorrhage following injury
Readcode	S63z.00	Other cerebral haemorrhage following injury NOS
ICD-10	G458	Other transient cerebral ischaemic attacks and related syndromes
ICD-10	G459	Transient cerebral ischaemic attack, unspecified
ICD-10	I690	Sequelae of subarachnoid haemorrhage
ICD-10	I61	Intracerebral haemorrhage
ICD-10	I60	Subarachnoid haemorrhage
ICD-10	I620	Subdural haemorrhage (acute)(nontraumatic)
ICD-10	I621	Nontraumatic extradural haemorrhage
ICD-10	I629	Intracranial haemorrhage (nontraumatic), unspecified
ICD-10	I693	Sequelae of cerebral infarction
ICD-10	I63	Cerebral infarction
ICD-10	I691	Sequelae of intracerebral haemorrhage
ICD-10	I692	Sequelae of other nontraumatic intracranial haemorrhage
ICD-10	I694	Sequelae of stroke, not specified as haemorrhage or infarction
ICD-10	I698	Sequelae of other and unspecified cerebrovascular diseases
ICD-10	G463	Brain stem stroke syndrome
ICD-10	G464	Cerebellar stroke syndrome
ICD-10	G465	Pure motor lacunar syndrome
ICD-10	G466	Pure sensory lacunar syndrome
ICD-10	G467	Other lacunar syndromes
ICD-10	I64	Stroke, not specified as haemorrhage or infarction
Valvular Heart Disease		
Readcode	791..00	Valves of heart and adjacent structures operations
Readcode	7910	Plastic repair of mitral valve
Readcode	7910.11	Mitral valvuloplasty

Readcode	7910.12	Replacement of mitral valve
Readcode	7910000	Allograft replacement of mitral valve
Readcode	7910100	Xenograft replacement of mitral valve
Readcode	7910200	Prosthetic replacement of mitral valve
Readcode	7910211	Bjork-Shiley prosthetic replacement of mitral valve
Readcode	7910212	Bjork-Shiley prosthetic replacement of mitral valve
Readcode	7910213	Carpentier prosthetic replacement of mitral valve
Readcode	7910214	Edwards prosthetic replacement of mitral valve
Readcode	7910300	Replacement of mitral valve NEC
Readcode	7910400	Mitral valvuloplasty NEC
Readcode	7910y00	Other specified plastic repair of mitral valve
Readcode	7910z00	Plastic repair of mitral valve NOS
Readcode	7911	Plastic repair of aortic valve
Readcode	7911.11	Aortic valvuloplasty
Readcode	7911.12	Replacement of aortic valve
Readcode	7911000	Allograft replacement of aortic valve
Readcode	7911100	Xenograft replacement of aortic valve
Readcode	7911200	Prosthetic replacement of aortic valve
Readcode	7911300	Replacement of aortic valve NEC
Readcode	7911400	Aortic valvuloplasty NEC
Readcode	7911y00	Other specified plastic repair of aortic valve
Readcode	7911z00	Plastic repair of aortic valve NOS
Readcode	7912	Plastic repair of tricuspid valve
Readcode	7912.11	Replacement of tricuspid valve
Readcode	7912.12	Tricuspid valvuloplasty
Readcode	7912000	Allograft replacement of tricuspid valve
Readcode	7912100	Xenograft replacement of tricuspid valve
Readcode	7912200	Prosthetic replacement of tricuspid valve
Readcode	7912300	Replacement of tricuspid valve NEC
Readcode	7912y00	Other specified plastic repair of tricuspid valve
Readcode	7912z00	Plastic repair of tricuspid valve NOS
Readcode	7913	Plastic repair of pulmonary valve
Readcode	7913.11	Pulmonary valvuloplasty
Readcode	7913.12	Replacement of pulmonary valve
Readcode	7913000	Allograft replacement of pulmonary valve
Readcode	7913100	Xenograft replacement of pulmonary valve
Readcode	7913200	Prosthetic replacement of pulmonary valve
Readcode	7913300	Replacement of pulmonary valve NEC
Readcode	7913400	Pulmonary valvuloplasty NEC
Readcode	7913y00	Other specified plastic repair of pulmonary valve
Readcode	7913z00	Plastic repair of pulmonary valve NOS
Readcode	7914	Plastic repair of unspecified valve of heart
Readcode	7914.11	Replacement of unspecified valve of heart
Readcode	7914000	Allograft replacement of valve of heart NEC
Readcode	7914100	Xenograft replacement of valve of heart NEC
Readcode	7914200	Prosthetic replacement of valve of heart NEC
Readcode	7914211	Edwards prosthetic replacement of valve of heart
Readcode	7914212	Starr prosthetic replacement of valve of heart
Readcode	7914300	Replacement of valve of heart NEC
Readcode	7914400	Valvuloplasty of heart NEC
Readcode	7914y00	Other specified plastic repair of unspecified valve of heart
Readcode	7914z00	Plastic repair of unspecified valve of heart NOS
Readcode	7915	Revision of plastic repair of valve of heart
Readcode	7915000	Revision of plastic repair of mitral valve
Readcode	7915100	Revision of plastic repair of aortic valve
Readcode	7915200	Revision of plastic repair of tricuspid valve
Readcode	7915300	Revision of plastic repair of pulmonary valve
Readcode	7915y00	Other specified revision of plastic repair of valve of heart

Readcode	7916.11	Open heart valvotomy
Readcode	7916000	Open mitral valvotomy
Readcode	7916100	Open aortic valvotomy
Readcode	7916200	Open tricuspid valvotomy
Readcode	7916300	Open pulmonary valvotomy
Readcode	7916z11	Heart valvotomy NEC
Readcode	7917.11	Closed heart valvotomy
Readcode	7917000	Closed mitral valvotomy
Readcode	7917100	Closed aortic valvotomy
Readcode	7917300	Closed pulmonary valvotomy
Readcode	7918000	Annuloplasty of mitral valve
Readcode	7919.11	Percutaneous transluminal valvotomy
Readcode	7919000	Percutaneous transluminal mitral valvotomy
Readcode	7919100	Percutaneous transluminal aortic valvotomy
Readcode	7919300	Percutaneous transluminal pulmonary valvotomy
Readcode	7919400	Percutaneous transluminal valvuloplasty
Readcode	791A200	Repair of subaortic stenosis
Readcode	791A300	Repair of supraaortic stenosis
Readcode	A932.11	Syphilitic valve disease
Readcode	G11..00	Mitral valve diseases
Readcode	G11..11	Rheumatic mitral valve disease
Readcode	G110.00	Mitral stenosis
Readcode	G110.11	Rheumatic mitral stenosis
Readcode	G111.00	Rheumatic mitral insufficiency
Readcode	G111.11	Mitral incompetence - rheumatic
Readcode	G111.12	Mitral regurgitation - rheumatic
Readcode	G112.00	Mitral stenosis with insufficiency
Readcode	G112.12	Mitral stenosis with incompetence
Readcode	G112.13	Mitral stenosis with regurgitation
Readcode	G113.00	Nonrheumatic mitral valve stenosis
Readcode	G114.00	Ruptured mitral valve cusp
Readcode	G11z.00	Mitral valve disease NOS
Readcode	G12..00	Rheumatic aortic valve disease
Readcode	G120.00	Rheumatic aortic stenosis
Readcode	G121.00	Rheumatic aortic insufficiency
Readcode	G121.11	Aortic incompetence - rheumatic
Readcode	G121.12	Aortic regurgitation - rheumatic
Readcode	G122.00	Rheumatic aortic stenosis with insufficiency
Readcode	G12z.00	Rheumatic aortic valve disease NOS
Readcode	G13..00	Diseases of mitral and aortic valves
Readcode	G130.00	Mitral and aortic stenosis
Readcode	G131.00	Mitral stenosis and aortic insufficiency
Readcode	G131.13	Mitral stenosis and aortic incompetence
Readcode	G131.14	Mitral stenosis and aortic regurgitation
Readcode	G132.00	Mitral insufficiency and aortic stenosis
Readcode	G132.12	Mitral incompetence and aortic stenosis
Readcode	G132.13	Mitral regurgitation and aortic stenosis
Readcode	G133.00	Mitral and aortic incompetence
Readcode	G133.11	Mitral and aortic insufficiency
Readcode	G133.12	Mitral and aortic regurgitation
Readcode	G13y.00	Multiple mitral and aortic valve involvement
Readcode	G13z.00	Mitral and aortic valve disease NOS
Readcode	G140.00	Tricuspid valve disease NEC
Readcode	G140000	Rheumatic tricuspid stenosis
Readcode	G140100	Rheumatic tricuspid insufficiency
Readcode	G140111	Tricuspid regurgitation - rheumatic
Readcode	G140112	Tricuspid incompetence - rheumatic
Readcode	G140200	Rheumatic tricuspid stenosis and insufficiency

Readcode	G14021X	Rheumatic tricuspid stenosis and regurgitation
Readcode	G14021Y	Rheumatic tricuspid stenosis and incompetence
Readcode	G140300	Tricuspid stenosis, cause unspecified
Readcode	G140400	Tricuspid insufficiency, cause unspecified
Readcode	G140412	Tricuspid incompetence, cause unspecified
Readcode	G140413	Tricuspid regurgitation, cause unspecified
Readcode	G140500	Tricuspid stenosis and insufficiency, cause unspecified
Readcode	G140514	Tricuspid stenosis and regurgitation, cause unspecified
Readcode	G140z00	Rheumatic tricuspid valve disease NOS
Readcode	G141.00	Rheumatic pulmonary valve disease
Readcode	G141000	Rheumatic pulmonary stenosis
Readcode	G141100	Rheumatic pulmonary insufficiency
Readcode	G141z00	Rheumatic pulmonary valve disease NOS
Readcode	G54..11	Heart valve disorders - non rheumatic
Readcode	G540.00	Mitral valve incompetence
Readcode	G540.12	Mitral valve insufficiency
Readcode	G540.14	Mitral valve regurgitation
Readcode	G540.15	Mitral valve prolapse
Readcode	G540.16	Mitral regurgitation
Readcode	G540000	Mitral incompetence, non-rheumatic
Readcode	G540100	Mitral incompetence, cause unspecified
Readcode	G540200	Mitral valve prolapse
Readcode	G540300	Mitral valve leaf prolapse
Readcode	G540z00	Mitral valve disorders NOS
Readcode	G541.00	Aortic valve disorders
Readcode	G541000	Aortic incompetence, non-rheumatic
Readcode	G541011	Aortic insufficiency, non-rheumatic
Readcode	G541012	Aortic regurgitation, non-rheumatic
Readcode	G541100	Aortic stenosis, non-rheumatic
Readcode	G541200	Aortic incompetence alone, cause unspecified
Readcode	G541211	Aortic insufficiency alone, cause unspecified
Readcode	G541212	Aortic regurgitation alone, cause unspecified
Readcode	G541300	Aortic stenosis alone, cause unspecified
Readcode	G541400	Aortic valve stenosis with insufficiency
Readcode	G541500	Aortic stenosis
Readcode	G541600	Aortic valve sclerosis
Readcode	G541z00	Aortic valve disorders NOS
Readcode	G542.00	Tricuspid valve disorders, non-rheumatic
Readcode	G542000	Tricuspid incompetence, non-rheumatic
Readcode	G542011	Tricuspid insufficiency, non-rheumatic
Readcode	G542012	Tricuspid regurgitation, non-rheumatic
Readcode	G542100	Tricuspid stenosis, non-rheumatic
Readcode	G542200	Nonrheumatic tricuspid valve stenosis with insufficiency
Readcode	G542X00	Nonrheumatic tricuspid valve disorder, unspecified
Readcode	G542z00	Tricuspid valve disorders NOS
Readcode	G543.00	Pulmonary valve disorders
Readcode	G543000	Pulmonary incompetence, non-rheumatic
Readcode	G543011	Pulmonary insufficiency, non-rheumatic
Readcode	G543012	Pulmonary regurgitation, non-rheumatic
Readcode	G543100	Pulmonary stenosis, non-rheumatic
Readcode	G543200	Pulmonary incompetence, cause unspecified
Readcode	G543213	Pulmonary insufficiency, cause unspecified
Readcode	G543215	Pulmonary regurgitation, cause unspecified
Readcode	G543300	Pulmonary stenosis, cause unspecified
Readcode	G543311	Pulmonary stenosis, cause unspecified
Readcode	G543400	Pulmonary valve stenosis with insufficiency
Readcode	G543z00	Pulmonary valve disorders NOS
Readcode	G544.00	Multiple valve diseases

Readcode	G544000	Disorders of both aortic and tricuspid valves
Readcode	G544100	Disorders of both mitral and tricuspid valves
Readcode	G544200	Combined disorders of mitral, aortic and tricuspid valves
Readcode	G544X00	Multiple valve disease, unspecified
Readcode	G54z000	Incompetence of unspecified heart valve
Readcode	G54z013	Regurgitation of unspecified heart valve
Readcode	G54z014	Insufficiency of unspecified heart valve
Readcode	G54z100	Stenosis of unspecified heart valve
Readcode	G54z500	Valvular heart disease
Readcode	Gyu1000	[X]Other mitral valve diseases
Readcode	Gyu1100	[X]Other rheumatic aortic valve diseases
Readcode	Gyu5500	[X]Other nonrheumatic mitral valve disorders
Readcode	Gyu5600	[X]Other aortic valve disorders
Readcode	Gyu5800	[X]Other pulmonary valve disorders
Readcode	Gyu5A00	[X]Aortic valve disorders in diseases classified elsewhere
Readcode	P602.00	Congenital pulmonary stenosis
Readcode	P602z00	Congenital pulmonary stenosis NOS
Readcode	P61..00	Congenital tricuspid atresia and stenosis
Readcode	P611.00	Congenital tricuspid stenosis
Readcode	P62..00	Ebstein's anomaly
Readcode	P63..00	Congenital aortic valve stenosis
Readcode	P64..00	Congenital aortic valve insufficiency
Readcode	P640.00	Congenital aortic valve insufficiency, unspecified
Readcode	P641.00	Bicuspid aortic valve
Readcode	P64z.00	Congenital aortic valve insufficiency NOS
Readcode	P65..00	Congenital mitral stenosis
Readcode	P650.00	Congenital mitral stenosis, unspecified
Readcode	P66..00	Congenital mitral insufficiency
Readcode	P6W..00	Congenital malformation of aortic and mitral valves unsp
Readcode	P6X..00	Congenital malformation of tricuspid valve, unspecified
Readcode	P6y0.00	Subaortic stenosis
Readcode	P6y2.00	Pulmonary infundibular stenosis
Readcode	P712.13	Postductal aortic stenosis
Readcode	P722400	Supra-valvular aortic stenosis
ICD-10	I05	Rheumatic mitral valve diseases
ICD-10	I06	Rheumatic aortic valve diseases
ICD-10	I07	Rheumatic tricuspid valve diseases
ICD-10	I08	Multiple valve diseases
ICD-10	I34	Nonrheumatic mitral valve disorders
ICD-10	I35	Nonrheumatic aortic valve disorders
ICD-10	I36	Nonrheumatic tricuspid valve disorders
ICD-10	I37	Pulmonary valve disorders
ICD-10	Q22	Congenital pulmonary valve stenosis
ICD-10	Q22.1	Congenital pulmonary valve stenosis
ICD-10	Q22.2	Congenital pulmonary valve insufficiency
ICD-10	Q22.3	Other congenital malformations of pulmonary valve
ICD-10	Q22.4	Congenital tricuspid stenosis
ICD-10	Q22.5	Ebstein's anomaly
ICD-10	Q22.8	Other congenital malformations of tricuspid valve
ICD-10	Q22.9	Congenital malformation of tricuspid valve, unspecified
ICD-10	Q23	Congenital stenosis of aortic valve
ICD-10	Q23.0	Congenital stenosis of aortic valve
ICD-10	Q23.1	Congenital insufficiency of aortic valve
ICD-10	Q23.2	Congenital mitral stenosis
ICD-10	Q23.3	Congenital mitral insufficiency
ICD-10	Q23.8	Other congenital malformations of aortic and mitral valves
ICD-10	Q23.9	Congenital malformation of aortic and mitral valves, unspecified

Supplementary Figure 1. Study design process leading to selection of study outcomes

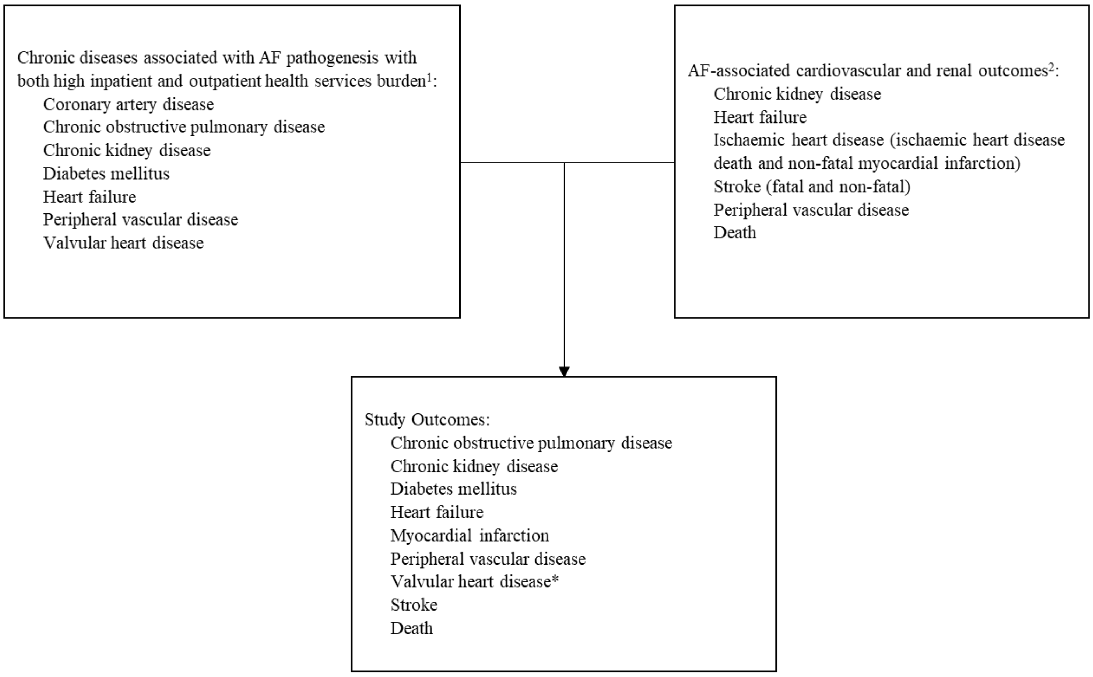

[1] Hindricks G, Potpara T, Dagres N, et al. 2020 ESC Guidelines for the diagnosis and management of atrial fibrillation developed in collaboration with the European Association for Cardio-Thoracic Surgery (EACTS) The Task Force for the diagnosis and management of atrial fibrillation of the European Society of Cardiology (ESC) Developed with the special contribution of the European Heart Rhythm Association (EHRA) of the ESC. 2021;42(5):373-498.
[2] Odutayo A, Wong CX, Hsiao AJ, et al. Atrial fibrillation and risks of cardiovascular disease, renal disease, and death: systematic review and meta-analysis. *BMJ* 2016;354

* Aortic stenosis was further specified in addition to valvular heart disease given the increasing availability and randomised controlled trial evidence for earlier treatment, and increasing therapeutic options across operative risk profiles (Vahanian A, Beyersdorf F, Praz F, et al. 2021 ESC/EACTS Guidelines for the management of valvular heart disease: developed by the Task Force for the management of valvular heart disease of the European Society of Cardiology (ESC) and the European Association for Cardio-Thoracic Surgery (EACTS). *Eur Heart J* 2022;43(7):561-632.)

Supplementary Results

Supplementary Table 4. Baseline characteristics of testing set, stratified by incident AF and predicted AF risk

	**Incident atrial fibrillation**		**FIND-AF predicted risk**	
	**no AF**<br>**n (%)**	**AF**<br>**n (%)**	**Lower risk**<br>**n (%)**	**Higher risk**<br>**n (%)**
	414 676	1 552	333 286	82 942
Demographics				
Age, years	49·82 (15·38)	73·87 (12·47)	44·11 (10·40)	73·24 (8·75)
Sex (women)	210 646 (50·8)	755 (48·6)	170 568 (51·2)	41 210 (49·7)
Ethnicity				
Asian	8 258 (2·0)	21 (1·5)	7 385 (2·2)	894 (1·1)
Black	6 390 (1·5)	9 (0·6)	5 786 (1·7)	613 (0·7)
Other	27 805 (6·7)	106 (7·4)	22 033 (6·6)	5 878 (7·1)
Unknown	93 630 (22·6)	36 (2·5)	91 505 (27·5)	2 161 (2·6)
White	278 714 (67·2)	1 259 (88·0)	206 577 (62·0)	73 396 (88·5)
Comorbidities				
Diabetes mellitus	14 649 (3·5)	171 (11·0)	6328 (1·9)	8072 (9·7)
Stroke or TIA	7 467 (1·8)	189 (12·2)	1376 (0·4)	6375 (7·7)
Ischaemic heart disease	15 483 (3·7)	314 (20·2)	3299 (1·0)	12486 (15·1)
Hypertension	49 494 (11·9)	621 (40·0)	20139 (6·0)	29594 (35·7)
Heart failure	2 745 (0·7)	132 (8·5)	163 (0·0)	2748 (3·3)
Dyslipidaemia	12 122 (2·9)	121 (7·8)	6095 (1·8)	5984 (7·2)
Hyperthyroidism	3 203 (0·8)	44 (2·8)	1883 (0·6)	1370 (1·7)

COPD	4 987 (1·2)	106 (6·8)	1111 (0·3)	4019 (4·8)
Chronic kidney disease	5 839 (1·4)	99 (6·4)	2938 (0·9)	2990 (3·6)
Anaemia	13 165 (3·2)	106 (6·8)	9118 (2·7)	4251 (5·1)
Cancer	14 710 (3·5)	186 (12·0)	6120 (1·8)	8303 (10·0)
Valvular heart disease	1 881 (0·5)	84 (5·4)	562 (0·2)	1414 (1·7)
Mean CHA_2DS_2-VASc score (SD)	0·97 (1·03)	2·74 (1·40)	0·62 (0·62)	2·42 (1·14)

AF, atrial fibrillation; CHA_2DS_2-VASc, Congestive heart failure, Hypertension, Age >75 years [2 points], Stroke/transient ischemic attack/thromboembolism [2 points], Vascular disease, Age 65-74 years, Sex Category; COPD, chronic obstructive pulmonary disease; TIA, transient ischaemic attack

Wu J, *et al. Open Heart* 2023; 10:e002357. doi: 10.1136/openhrt-2023-002357

Supplement Table 5. Cumulative incidence rate for the 10 outcomes at 1, 5, and 10 years of follow up stratified by predicted AF risk, when incident AF cases are excluded.

	Cumulative incidence (per 1000 persons)					
Outcome	Predicted lower risk			Predicted higher risk		
	1-year	5-year	10-year	1-year	5-year	10-year
Aortic stenosis	0.1 (0.1-0.1)	0.5 (0.4-0.5)	1.2 (1.1-1.4)	1.5 (1.2-1.7)	7.2 (6.5-7.8)	16.4 (15.2-17.7)
COPD	31.8 (31.2-32.4)	125.8 (124.6-127.1)	219.3 (217.5-221.0)	67.7 (65.8-69.5)	241.4 (237.9-244.8)	389.9 (385.3-394.4)
Chronic kidney disease	2.3 (2.1-2.4)	10.5 (10.1-10.9)	34.1 (33.3-34.9)	17.8 (16.8-18.8)	82.2 (80.0-84.4)	236.4 (232.2-240.5)
Diabetes mellitus	7.1 (6.8-7.4)	25.8 (25.2-26.4)	57.0 (56.0-58.1)	18.3 (17.2-19.3)	64.8 (62.7-66.8)	121.4 (118.2-124.5)
Heart failure	0.4 (0.4-0.5)	2.6 (2.4-2.7)	7.2 (6.8-7.6)	9.9 (9.2-10.6)	49.3 (47.5-51.0)	102.3 (99.4-105.1)
Myocardial infarction	0.8 (0.7-0.9)	5.1 (4.8-5.4)	12.9 (12.4-13.4)	5.3 (4.7-5.8)	30.2 (28.7-31.7)	66.6 (64.0-69.1)
Peripheral vascular disease	0.4 (0.3-0.4)	1.9 (1.7-2.1)	5.6 (5.2-5.9)	3.8 (3.3-4.2)	19.7 (18.6-20.9)	42.5 (40.6-44.5)
Stroke/TIA	0.8 (0.7-0.9)	4.7 (4.4-4.9)	12.2 (11.8-12.7)	8.8 (8.1-9.5)	51.9 (50.0-53.7)	111.4 (108.3-114.4)
Valvular heart disease	0.4 (0.3-0.5)	1.6 (1.5-1.8)	4.0 (3.7-4.3)	2.5 (2.2-2.9)	13.0 (12.1-13.9)	29.8 (28.2-31.5)
All-cause mortality		9.2 (8.8-9.6)	27.9 (27.2-28.6)		130.3 (127.6-132.9)	287.1 (283.1-291.1)

The cumulative incidence rates do not significantly change compared with when incident AF cases during follow up are not excluded.

Wu J, *et al. Open Heart* 2023; 10:e002357. doi: 10.1136/openhrt-2023-002357

Supplementary Figure 2. Kaplan-Meier plots for incident outcomes in individuals aged 30-64 years at baseline

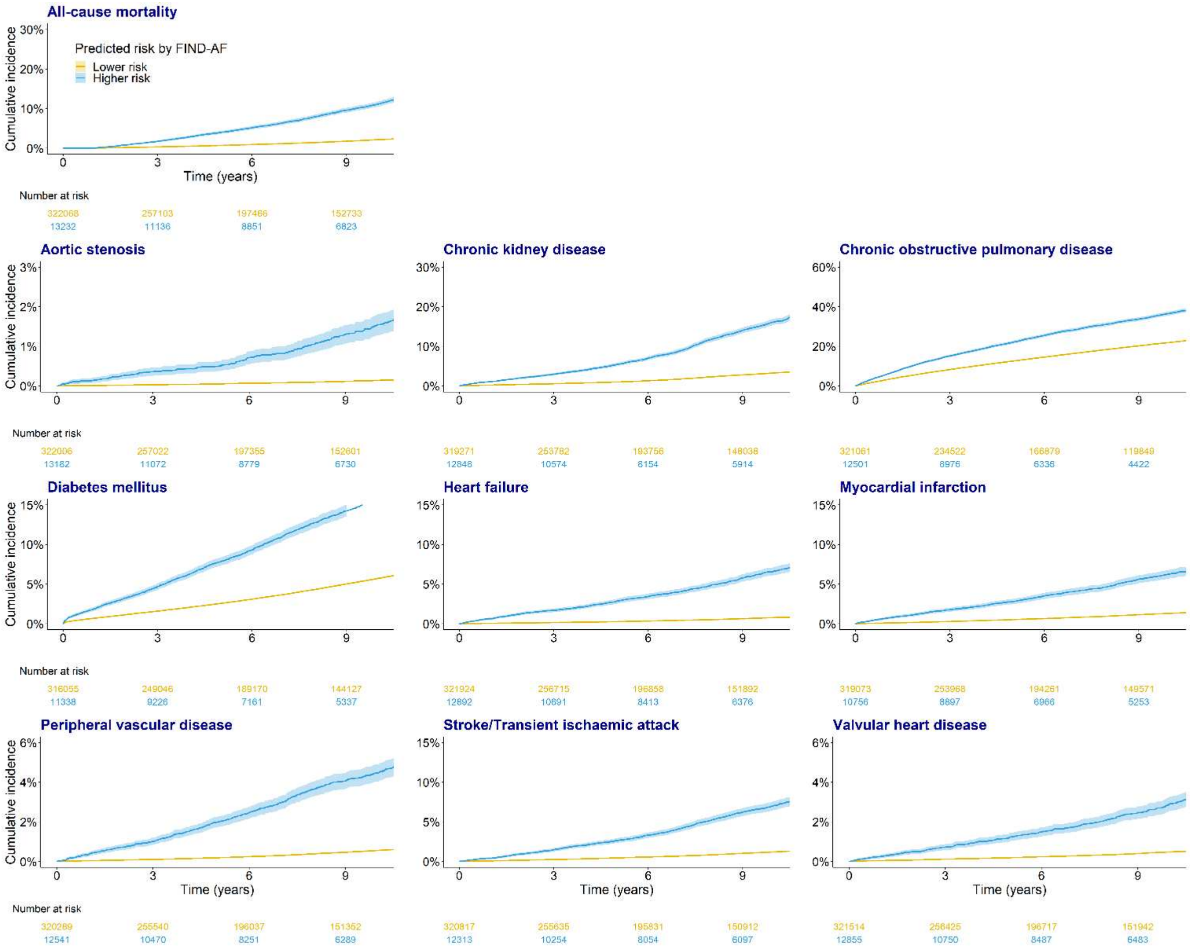

This figure demonstrates the Kaplan-Meier plots for the 10 outcomes investigated in the study in individuals aged 30-64 years at baseline. It maps the cumulative incidence on the y-axis and the follow up duration on the x-axis. The higher predicted AF risk cohort had a higher cumulative incidence for all outcomes compared with the lower predicted risk cohort.

Supplementary Figure 3. Kaplan-Meier plots for incident outcomes in individuals aged ≥65 years at baseline

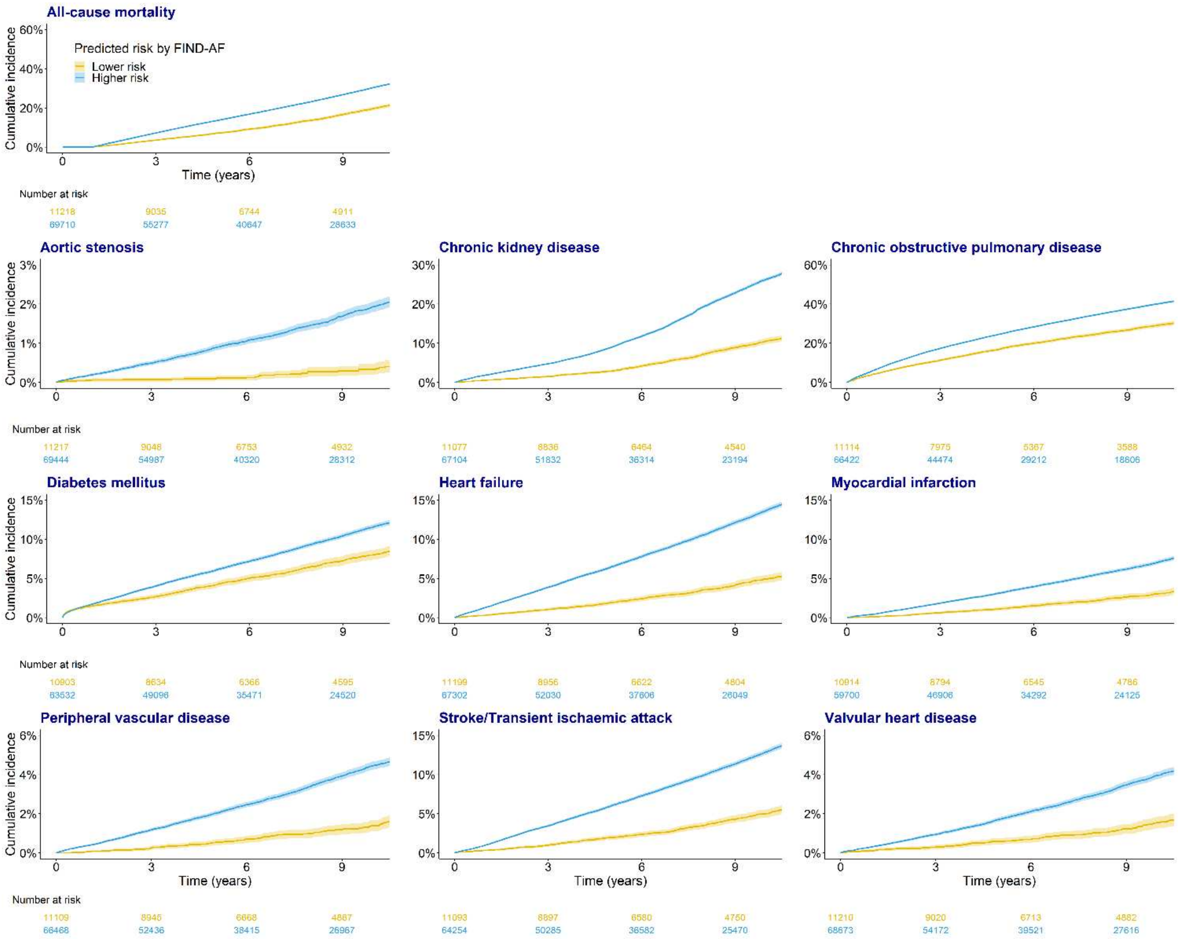

BMJ Publishing Group Limited (BMJ) disclaims all liability and responsibility arising from any reliance placed on this supplemental material which has been supplied by the author(s)

This figure demonstrates the Kaplan-Meier plots for the 10 outcomes investigated in the study in individuals aged ≥65 years at baseline. It maps the cumulative incidence on the y-axis and the follow up duration on the x-axis. The higher predicted AF risk cohort had a higher cumulative incidence for all outcomes compared with the lower predicted risk cohort.

Wu J, *et al. Open Heart* 2023; 10:e002357. doi: 10.1136/openhrt-2023-002357

Supplementary Figure 4. Kaplan-Meier plots for incident outcomes for men

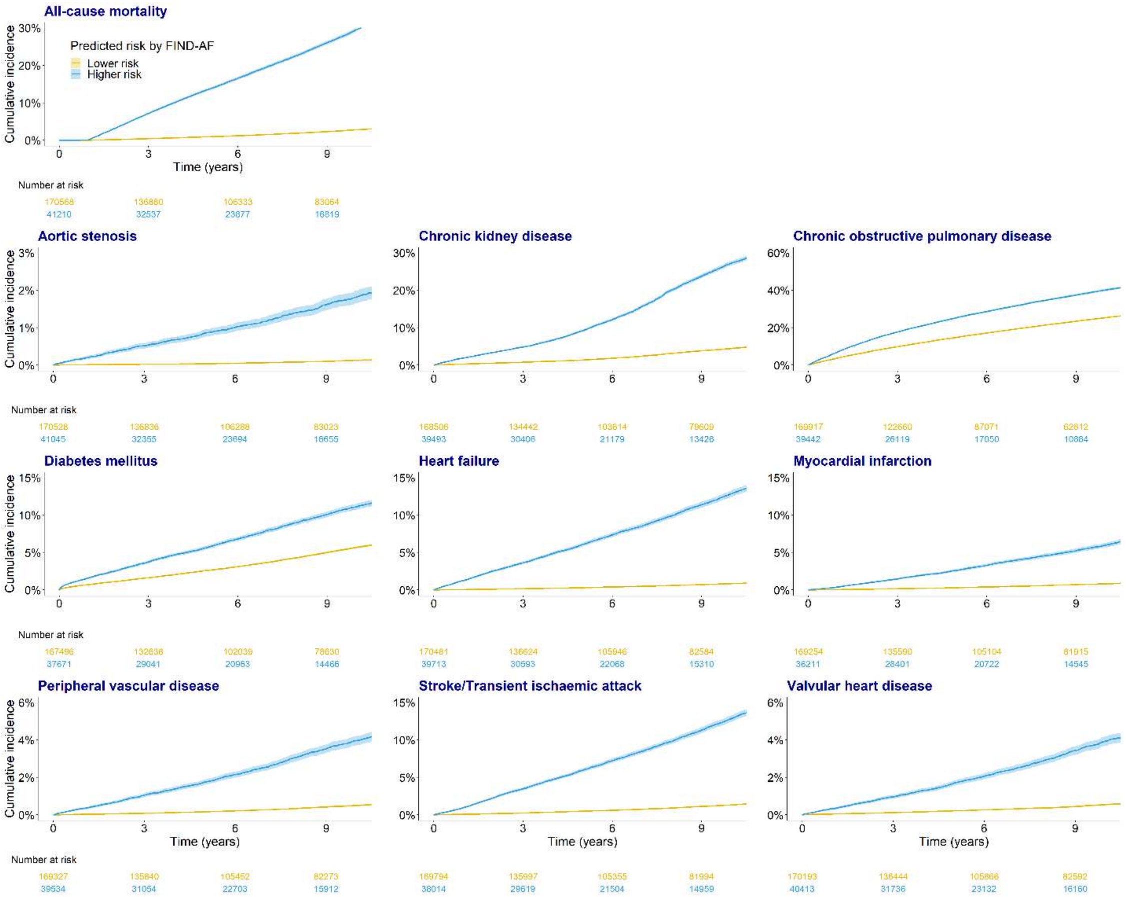

This figure demonstrates the Kaplan-Meier plots for the 10 outcomes investigated in the study in men. It maps the cumulative incidence on the y-axis and the follow up duration on the x-axis. The higher predicted AF risk cohort had a higher cumulative incidence for all outcomes compared with the lower predicted risk cohort.

Supplementary Figure 5. Kaplan-Meier plots for incident outcomes for women

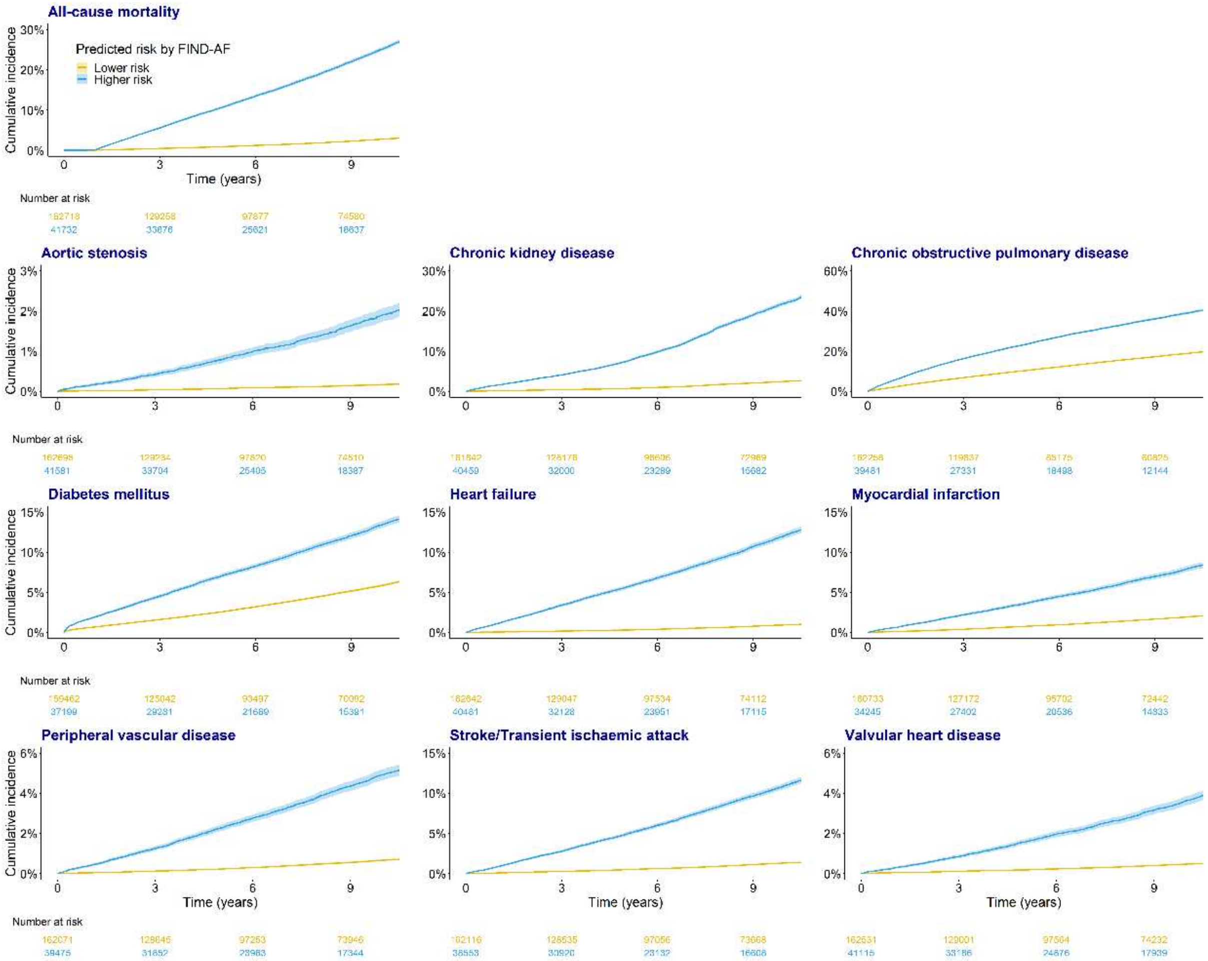

Wu J, *et al. Open Heart* 2023; 10:e002357. doi: 10.1136/openhrt-2023-002357

This figure demonstrates the Kaplan-Meier plots for the 10 outcomes investigated in the study in women. It maps the cumulative incidence on the y-axis and the follow up duration on the x-axis. The higher predicted AF risk cohort had a higher cumulative incidence for all outcomes compared with the lower predicted risk cohort.
